# Unraveling the pyroptosis-mitophagy axis: from molecular mechanisms to therapeutic opportunities

**DOI:** 10.3389/fimmu.2026.1950578

**Published:** 2026-10-13

**Authors:** Jiaqi Ban, Ranxing Chen, Lihong Ao, Hua Zhao, Jun Li, Yungeng Wei, Guichuan Huang

**Affiliations:** 1 School of Public Health, the Key Laboratory of Environmental Pollution Monitoring and Disease Control, Ministry of Education, Guizhou Medical University, Guizhou, China; 2 State Key Laboratory of Respiratory Disease, National Clinical Research Center for Respiratory Disease, National Center for Respiratory Medicine, Guangzhou Institute of Respiratory Health, The First Affiliated Hospital of Guangzhou Medical University, Guangzhou, China; 3 Department of Respiratory and Critical Care Medicine, Affiliated Hospital of Zunyi Medical University, Zunyi, Guizhou, China

**Keywords:** mitophagy, natural small molecules, NLRP3 inflammasomes, PINK1/Parkin pathway, pyroptosis, traditional Chinese medicine

## Abstract

Mitophagy and pyroptosis are central regulators of cell fate and inflammation, and they form a complex bidirectional network across many diseases. Mitophagy suppresses NLRP3 inflammasome activation by removing damaged mitochondria and thereby reducing mtROS and mtDNA release, which blocks pyroptosis. Conversely, pyroptosis can inhibit mitophagy by releasing inflammatory mediators and activating caspase-1, creating reinforcing or counterbalancing feedback loops. Signaling modules such as PINK1/Parkin, NOD2-RIPK2, Nrf2/HO-1, and AMPK critically modulate both mitophagy and pyroptosis. These processes interact broadly in organ injuries of the brain, liver, kidney, lung, bone, and heart to determine inflammation intensity and cell survival. Natural small molecules and traditional Chinese medicine that target the mitophagy–pyroptosis axis can modify pathological states including neurodegeneration, metabolic inflammation, and immune evasion, and thus represent a promising strategy for precision therapy.

## 1 Introduction

Mitochondria serve as the central hub for oxidative phosphorylation, facilitating ATP production, while their roles extend well beyond energy generation. Recent studies have demonstrated that damaged mitochondria release mitochondrial reactive oxygen species (mtROS), mitochondrial DNA (mtDNA), and various metabolic intermediates, all of which are directly involved in critical cellular processes, including inflammation, differentiation, and immune response (1–4). In the context of various diseases, mitochondrial damage not only exacerbates the energy crisis but also intensifies the inflammatory cascade through damage-associated molecular patterns (DAMPs). To preserve network homeostasis, cells initiate selective autophagy, known as mitophagy, to eliminate damaged mitochondria. This process is meticulously regulated by the PTEN-induced kinase 1/Parkin-mediated mitophagy (PINK1/Parkin) pathway and BCL2/adenovirus E1B 19 kDa protein-interacting protein 3/Nip3-like protein X-mediated mitophagy (BNIP3/NIX) pathway, along with a series of ubiquitin-receptor-mediated mechanisms, which collectively determine the dynamic equilibrium of mitochondrial quantity and quality (5). When mitophagy is compromised and dysfunctional mitochondria accumulate, excess mtROS and released mtDNA can activate inflammasomes such as NOD-like receptor family pyrin domain containing 3 (NLRP3), subsequently driving pyroptosis (6–9). Importantly, mitophagy plays a dual role in regulating cell death (10). Excessive cell death is associated with numerous neurodegenerative diseases, including Alzheimer’s disease and Parkinson’s disease, while inhibition of cell death can promote autoimmune disorders and tumor immune evasion. Consequently, targeting mitochondria to modulate cell death in various diseases has emerged as a cutting-edge strategy for therapeutic intervention (10, 11).

Pyroptosis is a lytic form of programmed cell death. The term ‘pyroptosis’ was first coined by Cookson and Brennan in 2001 to describe caspase-1-dependent macrophage death during bacterial infection, building upon earlier observations by Friedlander (1986) of anthrax lethal toxin-induced macrophage swelling and rupture (12, 13). Subsequent investigations confirmed that inflammasomes activate caspase-1, which cleaves pro-IL-1β and initiates cell death (14). Initially, it was believed that pyroptosis solely depended on caspase-1; however, recent findings have demonstrated that human caspase-4/5 and murine caspase-11 can also cleave Gasdermin-D (GSDMD), releasing its N-terminal fragment and forming pores in the plasma membrane. This ultimately results in membrane rupture and the leakage of interleukin-1β (IL-1β) and interleukin-18 (IL-18) (15). Under physiological conditions, this mechanism serves to eliminate intracellular pathogens. However, when overactivated, it can contribute to disease processes such as sepsis, atherosclerosis, and multi-organ fibrosis through a “cytokine storm”. Notably, caspase-1 can directly cleave the mitophagy core protein PINK1, thereby inhibiting the clearance of damaged mitochondria (16). This suggests that pyroptotic signaling can disrupt the regulation of mitochondrial quality control. Conversely, mitophagy can clear mtROS and mtDNA by removing damaged mitochondria, indicating that the maintenance of mitochondrial homeostasis may mitigate excessive activation of inflammasome and subsequent pyroptosis (17). Pyroptosis and mitophagy have a bidirectional relationship, forming a feedback loop with positive and negative interactions that collectively impact inflammation and cellular outcomes. This paper systematically outlines the bidirectional regulatory loops between mitophagy and pyroptosis—encompassing PINK1/Parkin, BNIP3/NIX, NOD2-RIPK2, Nrf2/HO-1, and AMPK signaling modules—and evaluates the spatiotemporal determinants that distinguish protective mitophagy from pathological pyroptosis, providing a framework for deploying specific therapeutics (e.g., NLRP3 inhibitor MCC950, PINK1 agonist urolithin A) at optimal disease stages for inflammatory conditions.

## 2 Mitophagy

Mitophagy serves as a critical quality control mechanism that enables cells to eliminate damaged or dysfunctional mitochondria through selective autophagy processes. Its primary role is to sustain the homeostatic balance between mitochondrial quantity and functionality, thereby ensuring the stability of the intracellular environment (18). This concept was initially introduced by Lemasters in 2005 (19). Mitophagy involves various crucial stages: autophagosome formation, selective identification and engulfment of impaired mitochondria, fusion of autophagosomes with lysosomes to generate autolysosome, and degradation of mitochondria inside these autolysosome (20). Mitochondria are the principal sites of ATP production in cells, supplying over 90% of cellular energy requirements (21). The integrity and repair capacity of mtDNA can be compromised during stress responses, such as depolarization of mitochondrial membrane potential (MMP, Δψm) and oxidative stress. When damage exceeds a certain threshold, dysfunctional mitochondria are removed via the autophagy pathway, a process referred to as mitophagy (6, 8). Basal levels of mitophagy are vital for maintaining cellular homeostasis, as they effectively mitigate cytotoxicity induced by damaged mitochondria by dynamically regulating mitochondrial numbers to align with cellular metabolic demands (22). Under stress conditions, the mitophagy pathway is markedly activated as an early adaptive response, facilitating cell survival by expediting the clearance of damaged mitochondria.

In recent years, numerous studies have demonstrated that moderate activation of mitophagy holds significant therapeutic potential for various diseases, including atherosclerosis (23), acute kidney injury (AKI) (24), and traumatic brain injury (25). This evidence further underscores the close association between mitophagy and the onset and progression of human diseases (26). Currently, the molecular mechanisms underlying mitophagy have been systematically elucidated and can be primarily categorized into two major pathways: Parkin RBR E3 ubiquitin protein ligase (PRKN, commonly referred to as Parkin)-dependent and non-dependent pathways (27). Among these, the PRKN-dependent pathway has received the most attention (28). The ubiquitin-dependent pathway, primarily mediated by PINK1 and Parkin, responds to mitochondrial depolarization by initiating the ubiquitination of outer mitochondrial membrane proteins, thereby recruiting autophagic machinery for mitochondrial clearance (28, 29). The receptor-mediated pathway utilizes proteins such as BNIP3, NIX, and FUNDC1, which directly interact with LC3/GABARAP family proteins via the LC3-interacting region (LIR) motif to facilitate mitochondrial clearance (30). Additionally, Autophagy-related protein 32 (Atg32) serves as a crucial receptor for mitophagy, dynamically regulating the interaction between Autophagy-related protein 8 (Atg8) and Autophagy-related protein 11 (Atg11), thereby influencing autophagosome formation (31–33). The endoplasmic reticulum (ER) plays a role in establishing mitochondria-ER contact sites through the mitochondria-ER encounter structure, the Get1/2 complex, and phospholipid methyltransferase Opi3, which provide membrane sources for autophagosomes and promote their assembly (34, 35). The ubiquitin-dependent pathway is further fine-tuned by regulatory molecules: TBK1 phosphorylates autophagy adaptors such as OPTN to amplify autophagy signaling (36), while deubiquitinating enzymes including USP30 and USP15 remove ubiquitin chains from mitochondrial proteins to prevent excessive mitophagy (37–39).Dysregulation of any of these pathways can result in abnormal mitophagy, leading to the accumulation of dysfunctional mitochondria, which in turn drives the onset and progression of mitochondria-related diseases (39).

## 3 Pyroptosis

Pyroptosis is a GSDMD-mediated form of programmed cell death characterized by both membrane lysis and pro-inflammatory effects, exhibiting distinct morphological and biochemical features compared to apoptosis (40–44). This process is characterized by plasma membrane perforation, cell swelling, osmotic lysis, and the abrupt release of substantial amounts of pro-inflammatory mediators, including IL-1β, IL-18, and High-mobility group box 1 (HMGB1). Additionally, it involves organelle deformation, DNA fragmentation, and nuclear condensation (16, 45). Cells undergoing pyroptosis exhibit DNA fragmentation, rendering them positive for both Terminal deoxynucleotidyl transferase dUTP nick-end labeling (TUNEL) and propidium iodide staining (46). Pyroptosis was initially identified in a bacterial infection model, where invasive pathogens induce macrophage death in a process dependent on caspase-1 (47, 48). As the Gasdermin (GSDM) family (GSDMA-E and DFNB59) was systematically characterized, the definition of pyroptosis evolved from “caspase-1 dependent” to “GSDM pore dependent” (49, 50). Current evidence indicates that Gasdermin-B (GSDMB), Gasdermin-C (GSDMC), GSDMD, and Gasdermin-E (GSDME) can directly form pores, while the functions of the remaining family members remain to be clarified. Five primary hypotheses have been proposed regarding the mechanisms of pyroptosis: (1) the caspase-1-dependent GSDMD-mediated pathway (classical pathway) (51); (2) the caspase-4/5/11-dependent GSDMD-mediated pathway (non-classical pathway) (52); (3) the caspase-3-dependent GSDME-mediated pathway (53); (4) the granzyme A-mediated GSDMB activation pathway (54); and (5) the GSDMC-mediated pathway (55).

In the classical pathway, inflammasome-activated caspase-1 cleaves GSDMD to generate its pore-forming N-terminal fragment (GSDMD-N), which forms membrane pores, leading to cell swelling, lysis, and the release of mature IL-1β and IL-18 (51, 56, 57). The non-classical pathway is mediated by caspase-4/5 in humans or caspase-11 in mice, which directly sense cytosolic LPS and cleave GSDMD to initiate pyroptosis (58, 59). Unlike caspase-1, caspase-11 does not directly process pro-IL-1β or pro-IL-18, but can activate the pannexin-1-P2X7 axis to amplify inflammatory signals (60, 61).

Pyroptosis is intricately regulated by multiple factors, primarily encompassing the following aspects: (1) Ionic balance: Elevation of intracellular calcium ion (Ca^2+^) promotes the activation of NLRP3 inflammasomes (62). The non-selective cation channel P2X7 mediates potassium ion (K^+^) efflux and also activates NLRP3 (63). Additionally, Transient receptor potential melastatin 7 (TRPM7)-mediated increases in zinc ion (Zn^2+^) influx can induce the generation of reactive oxygen species (ROS), which subsequently triggers pyroptosis (64). Changes in ionic homeostasis regulate pyroptosis by influencing inflammasome activity and membrane stability. (2) Signaling pathway interaction: The cyclic GMP–AMP synthase – stimulator of interferon genes (cGAS-STING) pathway links DNA damage to anti-tumor responses, with its sustained activation capable of inducing cell death and inhibiting tumorigenesis (65, 66). Activation of the Janus kinase 2/Signal transducer and activator of transcription 3 (JAK2/STAT3) pathway enhances the transcription of TRPM7 and NLRP3, thereby driving pyroptosis (62). Taletrectinib inhibits Protein kinase B/mechanistic target of rapamycin (AKT/mTOR) signaling by targeting Src family kinases (SRCs) and also promotes pyroptosis (67). Thus, pyroptosis is not an isolated event; rather, it is interconnected with multiple intracellular signaling networks. (3) Epigenetic modifications: Enhancer of zeste homolog 2 (EZH2) can methylate STAT3, enhancing its transcriptional activity, while inhibition of either EZH2 or STAT3 can upregulate proteins associated with pyroptosis (68). Sirtuin 1 (SIRT1) is positioned upstream of ROS, and bursts of ROS increase Tumor necrosis factor receptor-associated factor 6 (TRAF6) levels, promoting pyroptosis, whereas Sirtuin 3 (SIRT3) inhibits pyroptosis by reducing ROS levels (69). In small cell lung cancer, cytoplasmic chromatin fragments activate Tumor necrosis factor alpha-induced protein 3 (A20), which stabilizes NLRP3 inflammasomes through deubiquitination, thereby facilitating pyroptosis (70). Furthermore, DNA methylation inhibits Sp1 binding sites, suppressing GSDME expression and reducing pyroptosis (71). Collectively, these findings indicate that epigenetic modifications can influence pyroptosis by regulating gene expression and protein activity.

Pyroptosis encompasses both “execution” and “command” functions. The transition between these functions relies not only on the classical inflammasome-GSDMD axis but is also intricately regulated by various factors, including ion homeostasis, epigenetic modifications, signaling networks, and interactions among different modes of cell death. Inflammatory mediators such as IL-1β and HMGB1, released during pyroptosis, can influence neighboring cells, alter the tumor microenvironment, modulate stem cell characteristics, or contribute to damage in distant organs. The interplay of positive and negative feedback mechanisms positions pyroptosis as a central hub in the contexts of infection, inflammation, tumorigenesis, ischemia-reperfusion injury, and metabolic disorders.

## 4 Mechanism of interaction between pyroptosis and mitophagy

Emerging evidence suggests a relationship between pyroptosis and mitophagy in both human health and disease; however, specific mechanisms underlying their interaction remain poorly understood. The equilibrium within the pyroptosis-mitophagy pathway may serve to protect the host from inflammatory responses, thereby promoting overall health. Conversely, dysregulation of this pathway can result in pathological inflammatory responses that exacerbate mitochondrial damage and pyroptosis. In this section, we will summarize the potential mechanisms of interaction between pyroptosis and mitophagy (**Figure 1**).

**Figure 1:**
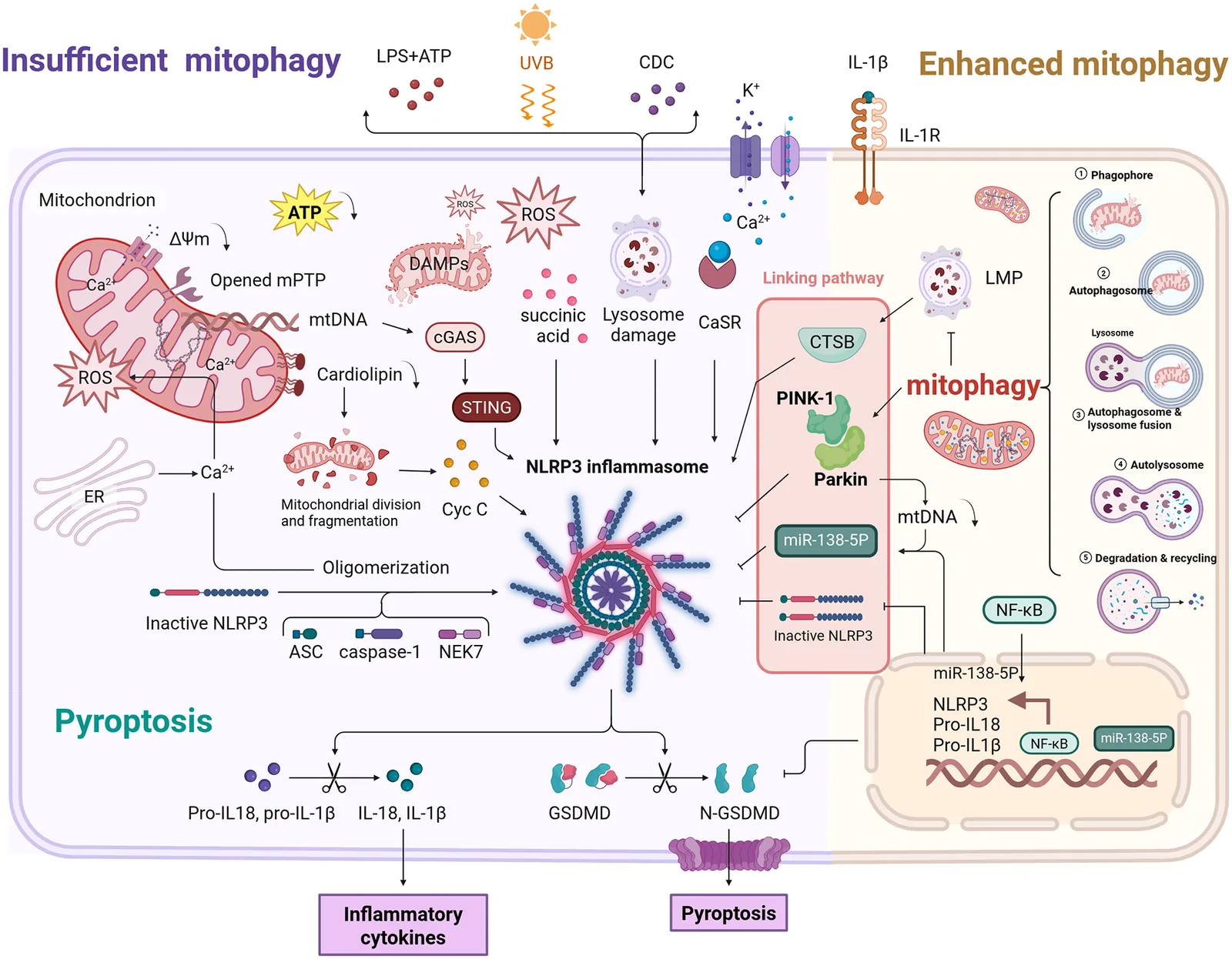
Mitophagy and pyroptosis constitute a core dynamic balance that determines cell fate. Pathological stimuli such as LPS+ATP, UVB, and rotenone induce mitochondrial damage, characterized by ΔΨm decline, ROS burst, mtDNA leakage, cardiolipin externalization, and mitochondrial fragmentation. These damage signals amplify inflammation through multiple pathways: mtDNA activates cGAS-STING; reduced cardiolipin promotes mitochondrial fission and cytochrome C release; succinate accumulation and Ca²^+^ signaling enhance NLRP3 activation. Ultimately, NLRP3 assembles with ASC and Caspase-1 to form the inflammasome, triggering IL-1β/IL-18 maturation and GSDMD-mediated membrane pore formation, driving pyroptosis. Conversely, PINK1/Parkin-mediated mitophagy clears damaged mitochondria and their ROS and mtDNA, eliminating NLRP3 activation signals at the source and inhibiting pyroptosis; it also reduces cytosolic mtDNA and elevates miR-138-5p to further suppress NLRP3. Notably, pyroptosis can trigger protective feedback: IL-1β enhances mitophagy; however, excessive mitophagy leads to lysosomal overload and LMP, releasing CTSB that directly activates NLRP3, reversing protective mitophagy into a pro-pyroptotic signal and forming a negative feedback loop that prevents excessive inflammatory amplification. This bidirectional regulation reveals how cells under stress balance mitochondrial quality control and inflammatory death to determine adaptation/repair or programmed demise.

### 4.1 Mechanism of mitophagy regulating pyroptosis

#### 4.1.1 Clearing damaged mitochondria and inhibiting ROS and mtDNA mediated activation of NLRP3 inflammasomes

Mitophagy, a crucial subtype of selective autophagy, preserves mitochondrial network homeostasis by facilitating the removal of damaged or excess mitochondria. This process inhibits inflammatory responses and programmed cell death mechanisms (72). Recent studies indicate that mitophagy is instrumental in regulating pyroptosis, primarily through the negative regulation of ROS and mtDNA release, which in turn obstructs the assembly and activation of NLRP3 inflammasomes. Mitochondria serve as the principal source of ROS within cells, particularly when dysfunction occurs in the electron transport chain (ETC) or when MMP declines (73). Under these conditions, ROS levels rise significantly, triggering oxidative stress responses. Numerous studies have demonstrated that exogenous stimuli, including ATP, monosodium urate (MSU) crystals, silica, and asbestos particles, can induce mitochondrial ROS accumulation, activate the NLRP3 inflammasome, and subsequently promote pyroptosis (74–76). For instance, in macrophages, ROS generated during the phagocytosis of silica and asbestos particles markedly enhances NLRP3 inflammasome formation and promotes the maturation and release of IL-1β and IL-18 (77). Conversely, the application of ROS inhibitors, such as N-acetyl-L-cysteine precursor (APDC), effectively prevents the activation of these inflammasomes and significantly diminishes the extent of pyroptosis (78). Further studies have demonstrated that rotenone, a mitochondrial complex I inhibitor, and antimycin A, a complex III inhibitor, can directly activate the NLRP3 inflammasome by inducing the production of ROS within mitochondria. This finding indicates that mtROS serve not only as byproducts of inflammatory responses but also as crucial upstream signaling molecules for NLRP3 activation (78). Similarly, in the context of AKI and sepsis-induced acute kidney injury (S-AKI), mitophagy mitigates pyroptosis by eliminating damaged mitochondria, thereby reducing mtROS production, inhibiting NLRP3 inflammasome activation, and decreasing pyroptosis (79).

Furthermore, mtDNA leakage, resulting from increased mitochondrial membrane permeability, has been identified as DAMPs that further amplify the inflammatory response by binding to the NLRP3 or cGAS-STING pathways (80, 81). Notably, mtDNA possesses a higher content of cytosine–phosphate–guanine (CpG) islands and greater oxidative sensitivity compared to nuclear DNA, making its release more likely to provoke an immune response (82). Mitophagy reduces cellular mtDNA leakage by eliminating damaged mitochondria, thus inhibiting NLRP3 inflammasome activation and pyroptosis. In sepsis-associated acute lung injury (S-ALI), Mitophagy decreases cytoplasmic mtDNA levels, leading to increased miR-138-5p expression. This directly targets NLRP3, inhibiting its activation and alleviating pyroptosis and pulmonary inflammation (83).

#### 4.1.2 Maintain mitochondrial homeostasis and regulate cellular energy metabolism

Ca^2+^ are crucial second messengers in intracellular signaling. Mitochondria play a key role in regulating cytoplasmic Ca^2+^ levels, but excessive Ca^2+^ can lead to mitochondrial dysfunction. Elevated cytosolic Ca^2+^ levels are consistently observed in the activation of the NLRP3 inflammasome in various stimuli, such as LPS+ATP, mycobacterial infection, UVB exposure, and cholesterol-dependent cytolysin exposure (84–87). Mechanistically, calcium-sensitive receptors directly interact with NLRP3, thereby amplifying inflammatory signals (88, 89). Furthermore, Ca^2+^ influx into the cytoplasm is a prevalent proximal event associated with NLRP3 inflammasome activation (89, 90).

Mitophagy is essential for cellular energy metabolism by controlling mitochondria quantity and function. Dysregulation can lead to pyroptosis through impacts on mitochondrial respiration. Inhibition of mitophagy can cause succinic acid accumulation, interacting with NLRP3 inflammasomes and affecting pyroptosis (91, 92). Atraric acid enhances mitophagy in chronic intermittent hypoxia models in mice and H9C2 cardiomyocytes by activating the Forkhead box protein O3a (FoxO3a)-PINK1/Parkin axis. This intervention restores ATP levels to 90% of control values within 24 hours and inhibits the activation of NLRP3 inflammasome, leading to a 60% decrease in caspase-1 activity and alleviating pyroptosis in cardiomyocytes (93).

#### 4.1.3 Maintain mitochondrial membrane potential and regulate mitochondrial dynamics

Mitophagy is essential for maintaining MMP. Impairment of mitophagy significantly reduces membrane potential, resulting in structural abnormalities and dysfunction of mitochondria (94). This loss of membrane potential not only disrupts ATP production but also facilitates the opening of mitochondrial permeability transition pores (mPTP), which subsequently initiates a cascade of pyroptosis (95–97). Furthermore, mitophagy influences mitochondrial dynamics by regulating the distribution and content of CL, a phospholipid exclusive to the inner mitochondrial membrane, when reduced, promotes mitochondrial division and exacerbates mitochondrial damage (98). The resulting imbalance between mitochondrial division and fusion leads to fragmentation, increases mitochondrial membrane permeability, and encourages the release of apoptotic factors such as cytochrome C. This release can further activate caspase family proteins, ultimately resulting in pyroptosis (99, 100). In a dry eye model, hyperosmolar stress maintained the mPTP in an open state, causing mtDNA to leak into the cytoplasm, where it was recognized by cGAS. Blockade of the stimulator of interferon genes-type I interferon (STING-IFN) axis significantly alleviated ocular surface inflammation (101).

### 4.2 Feedback regulation of pyroptosis on mitophagy

Pyroptosis is not only regulated by mitophagy, but also regulates mitophagy through a series of mechanisms, thus forming a negative feedback regulatory loop.

#### 4.2.1 Pyroptosis causes mitochondrial damage and triggers mitophagy

Activation of NLRP3 inflammasomes during pyroptosis leads to increased mitochondrial membrane permeabilization and loss of membrane potential, promoting pyroptosis and triggering mitophagy to remove damaged mitochondria. Excessive ROS production and lysosomal membrane permeability in pyroptosis induce mitophagy, which reduces NLRP3 inflammasome activation by lowering ROS levels and inflammatory factor release (16, 102). Some studies have also found that activated GSDMD-N first forms pores in the mitochondrial membrane, leading to mitochondrial damage, which occurs earlier than pore formation in the cell membrane (103). This feedback loop helps cells regulate inflammatory responses and cell death during stress to prevent excessive damage.

#### 4.2.2 Pyroptosis regulates mitophagy through inflammatory factors

During pyroptosis, mitophagy can be regulated by binding to specific receptors and activating downstream signaling (104). These factors significantly influence mitophagy in various disease models. IL-1β, a key inflammatory mediator released through GSDMD membrane pores during pyroptosis, classically initiates and amplifies inflammatory responses. Although mitophagy limits IL-1β release by suppressing pyroptosis, IL-1β released during pyroptosis may in turn feedback-regulate autophagic flux in a context-dependent manner. In LPS-induced ALI, IL-1β from pyroptosis triggers Myeloid differentiation primary response 88 (MyD88)-dependent signaling via IL-1R1, enhancing caspase-1-mediated pyroptosis. This leads to more IL-1β release, activating IL-1R1 in nearby macrophages, creating a positive feedback loop that worsens pulmonary inflammation (105). In the S-AKI model, mesenchymal stem cells (MSCs) that secrete tumor suppressor gene-6 (TSG-6) or upregulate SIRT1 lead to reduced Parkin acetylation, increased mitophagy, decreased mtDNA, and diminished pyroptosis (104). Additionally, in a copper-induced microglial pyroptosis model, copper exposure activates NLRP3 inflammasomes, resulting in the release of IL-1β and IL-18, which promote mitophagy and facilitate the clearance of damaged mitochondria (106).

#### 4.2.3 Inhibition of mitophagy by NLRP3 inflammasome activation

The NLRP3 inflammasome is a multiprotein complex whose activation initiates a cascade of intracellular signaling pathway alterations, thereby inhibiting mitophagy (107). The activation of caspase-1 by inflammasomes not only inhibits mitophagy but also exacerbates mitochondrial damage. Furthermore, K^+^ efflux serves as a common mechanism for NLRP3 inflammasome activation, while Ca^2+^ influx enhances NLRP3 activation by amplifying or synergizing K^+^ efflux signals. Recent research indicates that K^+^ efflux may precede Ca^2+^ influx during NLRP3 inflammasome activation (108–110), and highlights the critical role of the PINK1/Parkin pathway in maintaining mitochondrial homeostasis. However, activation of the NLRP3 inflammasome inhibits PINK1/Parkin-mediated mitophagy (111). Additionally, in the crystalline silica (CS) model, pyroptosis activates the NLRP3/caspase-1/GSDMD axis, leading to the degradation of PINK1/Parkin and the subsequent blockade of mitophagy, thereby creating a vicious cycle (112, 113).

### 4.3 Integrated spatiotemporal feedback model of the mitophagy-pyroptosis

The mitophagy-pyroptosis axis functions not as a linear pathway but as a dynamic bistable switch, with its output—cell survival versus inflammatory death—determined by the temporal integration of opposing signals that vary in dominance throughout disease progression. Mitophagy primarily suppresses pyroptosis by removing damaged mitochondria, thereby limiting the release of mtROS and mtDNA, which are critical upstream signals for NLRP3 inflammasome assembly (79, 83, 114–116). This negative regulation occurs via the PINK1/Parkin ubiquitin-dependent pathway, which responds to acute loss of ΔΨm, as well as through receptor-mediated pathways (BNIP3/NIX, FUNDC1) that directly engage LC3 via LIR motifs and are transcriptionally regulated under conditions of chronic hypoxia or metabolic stress (30, 117–120). Conversely, pyroptosis modulates mitophagy through a pro-pyroptotic positive feedback mechanism; sustained activation of NLRP3 and caspase-1 directly cleaves PINK1 and degrades Parkin, thereby abrogating mitophagy flux and establishing a self-perpetuating cycle of mitochondrial dysfunction and inflammation (16, 112, 121). This caspase-1-mediated blockade of mitophagy has been observed in crystalline silica-induced pulmonary fibrosis, contrast-induced AKI (112, 121).

Integrating the disparate signaling nodes into a cohesive framework reveals that miR-138-5p functions as a protective element that promotes mitophagy while directly targeting NLRP3 mRNA (83). Lysosomal degradative capacity serves as a critical determinant of whether mitophagic flux remains protective or becomes pathological. When mitophagic flux exceeds the processing capacity of lysosomes—as observed under high-fat conditions—lysosomal overload triggers lysosomal membrane permeabilization (LMP) and releases cathepsin B (CTSB) into the cytoplasm. Freed CTSB directly activates the NLRP3 inflammasome, shifting mitophagy from a protective process into a driver of pyroptosis (122, 123). Conversely, stabilizing lysosomal membranes and limiting CTSB leakage preserves productive mitophagic flux and suppresses NLRP3 activation (122, 123). Additionally, GSDMD pores formed during pyroptosis compromise mitochondrial membrane integrity, thereby exacerbating mitochondrial damage (103). The NLRP3/caspase-1 and PINK1/Parkin degradation axis represents the central positive feedback loop that sustains mitochondrial dysfunction and pyroptosis (16). In summary, the mitophagy-pyroptosis axis operates as an integrated bistable switch, wherein mitophagy inhibits pyroptosis through mitochondrial quality control (79, 83, 114, 115), while pyroptosis, in turn, obstructs mitophagy via caspase-1-mediated PINK1/Parkin degradation (112, 124). The overall outcome—protection versus inflammatory cell death—hinges on the temporal balance between these opposing mechanisms, with chronic activation of NLRP3/caspase-1 skewing the balance toward a self-reinforcing pathological cycle (112, 121, 124).

### 4.4 Key molecules and signaling pathways related to mitophagy and pyroptosis

#### 4.4.1 PINK1/Parkin signaling pathway

The PINK1/Parkin pathway regulates mitophagy by responding to decreased MMP. PINK1 stabilizes and accumulates on the translocase of the outer mitochondrial membrane (TOM) complex, recruiting Parkin. PINK1 activates Parkin’s E3 ubiquitin ligase function through Ser65-ubiquitin phosphorylation, leading to the ubiquitination of outer mitochondrial membrane proteins. This process generates a signal for autophagic recognition, allowing autophagy receptors to bind to LC3 for damaged mitochondria clearance (117, 118). The pathway has a narrow activity window. Insufficient activity can cause mtROS burst and mtDNA leakage, activating NLRP3 inflammasomes. Excessive activity can result in autophagy overload, leading to a switch to caspase-1/GSDMD-dependent autophagy-pyroptosis (125, 126).

Sestrin-2 (Sesn2) regulates anti-inflammatory protection: In the LPS-induced acute lung injury model, Sesn2 acts as a metabolic receptor regulating mitophagy through the PINK1/Parkin pathway. Sesn2 knockout in mice led to reduced PINK1 aggregation, inhibited Parkin translocation, decreased LC3B-II/LC3B-I ratio, Sequestosome 1 (p62) accumulation, and overactivation of the mtROS-NLRP3-ASC-Caspase-1 cascade. Elevated GSDMD-N, IL-1β, and IL-18 levels were observed in alveolar lavage fluid. AAV9-Sesn2 overexpression restored PINK1/Parkin function, reversed phenotypic changes, and reduced pulmonary edema and neutrophil infiltration (114).

**Transition from mitophagy to pyroptosis induced by ozone and hyperoxia in pro-inflammatory injury**

Ozone inhalation induces MMP depolarization by oxidizing CL, leading to PINK1 accumulation on the outer mitochondrial membrane and Parkin translocation peaking at 6 hours. This triggers excessive autophagic flux, depleting p62 and causing incomplete damaged mitochondria clearance. Subsequently, mtDNA release activates NLRP3 inflammasomes via mtROS synergy (127). Caspase-1 cleaves GSDMD, inducing pyroptosis in alveolar epithelial cells. In Parkinson’s disease models, continuous PINK1/Parkin-mediated mitophagy burdens lysosomal degradation, reducing membrane stability, increasing LMP, and releasing CTSB. Overactivated NLRP3 inflammasomes cleave and activate GSDMD, leading to pyroptosis and the release of inflammatory factors IL-1β and IL-18, forming a neuroinflammatory loop (127).

**Mitophagy inhibition as a strategy for AgNPs in nanotoxicology**

These nanoparticles, with a size of less than 20 nm, tend to accumulate in liver mitochondria due to limited clearance by the liver and kidneys. At low doses, AgNPs can facilitate the clearance of damaged mitochondrial networks through PINK1/Parkin-dependent mitophagy. Furthermore, they inhibit the formation of NLRP3-ASC spots and the release of IL-1β, thereby reducing pyroptosis in hepatocytes. In contrast, PINK1-knockout mice exhibited exacerbated lobular necrosis and elevated alanine aminotransferase (ALT)/aspartate aminotransferase (AST) levels, indicating that moderate mitophagy plays a protective role against the hepatotoxicity of AgNPs (128).

#### 4.4.2 NOD2-RIPK2 pathway

The nucleotide-binding oligomerization domain-containing protein 2/receptor-interacting serine/threonine-protein kinase 2 (NOD2-RIPK2) axis regulates NLRP3 inflammasome fate through S-acylation-lipid raft and Unc-51 like autophagy activating kinase 1 (ULK1)-Mitophagy mechanisms. NLRP3 is anchored to the Golgi network by Zinc finger DHHC-type palmitoyltransferase 7 (zDHHC7)-catalyzed S-acylation upon activation, facilitating ASC/pro-caspase-1 recruitment for assembly. NOD2 is S-acylated, incorporating into lipid rafts with RIPK2, activating TAK1-IKK-NF-κB and MKK4/7-JNK/p38 pathways. This modification exposes a p62-LIR-like motif, enabling NOD2 recognition by autophagosomes for degradation, preventing excessive inflammation. Inhibited autophagy leads to NOD2 accumulation, amplifying NF-κB/Mitogen-activated protein kinase (MAPK) signaling and inflammation (129). RIPK2 phosphorylates ULK1 at Ser555, enhancing mitophagy, reducing mtROS-NLRP3 axis activity, IL-1β release, and pyroptosis in microglia (130). Disruption of the NOD2-RIPK2 axis in THP-1 macrophages reduces mitophagy and increases NLRP3 inflammasome assembly. Restoring the ULK1-Ser555 phosphorylation site mutant counteracts AMP-activated protein kinase (AMPK)-ULK1 axis inhibition, decreasing ULK1 Ser555 phosphorylation levels, impairing Mitophagy flux, and promoting mtROS accumulation, NLRP3 inflammasome assembly, and IL-1β secretion. Over-expression of the phosphorylated mimic mutant ULK1-S555D reinstates mitophagy, inhibiting virus-induced NLRP3 inflammasome activation (130).

#### 4.4.3 Nuclear factor erythroid 2-related factor 2/heme oxygenase-1 signaling pathway (Nrf2/HO-1) pathway

Stimulation with Hydroquinone (HQ) in human retinal pigment epithelial cells (ARPE-19 cell line) induces a transient surge in ROS, triggering Nrf2 translocation and upregulation of antioxidant genes. HO-1 facilitates CL externalization via PINK1/Parkin-dependent pathway, promoting protective mitophagy. Prolonged stimulation leads to Nrf2/HO-1 exhaustion, shifting CL externalization to NLRP3 inflammasome, inducing pyroptosis. The Nrf2/HO-1 pathway acts as an “autophagy-pyroptosis” switch, determining CL fate (98). Rice bran extract under chronic cold stress enhances Nrf2 translocation, activates HO-1, reduces BNIP3, inhibits excessive mitophagy, downregulates NLRP3 and IL-1β, reversing pyroptosis in cardiomyocytes (131).

#### 4.4.4 The key role of AMPK

In a mouse model of spinal cord contusion (SCI), the Bexarotene (Bex) drug activates AMPK signaling to enhance autophagy and mitophagy, reducing oxidative stress and pyroptosis. Bex inhibits mTOR through AMPK, promoting transcription factor binding to IGHM enhancer 3 (TFE3) translocation. The AMPK-SKP2-CARM1 axis phosphorylates FOXO3a, inhibiting S-phase kinase-associated protein 2 (SKP2) and increasing coactivator-associated arginine methyltransferase 1 (CARM1) levels. This enhances TFE3 activity, upregulating mitophagy-related genes to clear damaged mitochondria and reduce ROS. Consequently, NLRP3 inflammasome activation is inhibited, blocking the pyroptosis pathway and reducing neuronal death for improved motor function recovery (132). Immunity-related GTPase M (IRGM) enhances AMPK activation by stabilizing p-AMPKThr172, facilitating ULK1 and Beclin1 phosphorylation for mitophagy and autophagosome formation. It interacts with Atg8/LC3 to enhance mitophagy efficiency, clearing mtROS and mtDNA to inhibit NLRP3 inflammasome assembly, caspase-1 activation, and IL-1β release, ultimately preventing neuronal pyroptosis (133). Furthermore, growth differentiation factor 11 (GDF11) in neurodegenerative disease models boosts TFE3-mediated autophagy, inhibiting pyroptosis via the AMPK- transient receptor potential mucolipin-1 (TRPML1)-Calcineurin pathway. GDF11 upregulates AMPK and TRPML1 activities, promoting mitophagy, reducing mitochondrial damage and ROS accumulation, and decreasing neuronal pyroptosis (134) (**Figure 2**).

**Figure 2:**
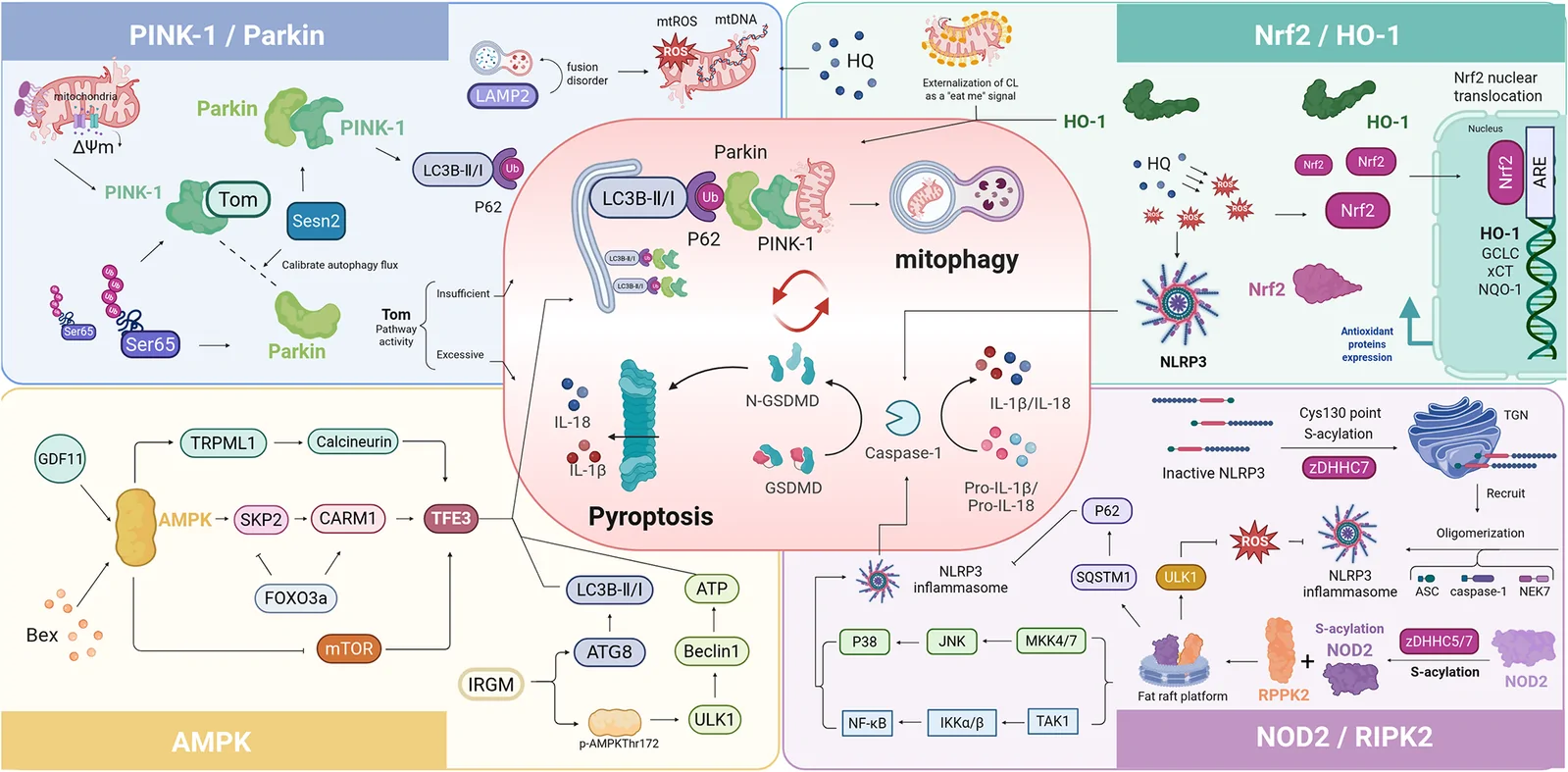
Signaling network integrating mitophagy and pyroptosis regulation. Four core pathways converge on the NLRP3 inflammasome to determine the balance between mitochondrial clearance and pyroptotic cell death. (1) PINK1/Parkin: Responds to ΔΨm loss and clears damaged mitochondria through Parkin-mediated ubiquitination. (2) NOD2-RIPK2: S-acylated NOD2 recruits RIPK2 to activate NF-κB/MAPK-driven inflammatory gene expression, while RIPK2 phosphorylates ULK1 to enhance mitophagy and restrain mtROS-NLRP3 signaling. (3) Nrf2/HO-1:Early Nrf2 activation upregulates HO-1 and antioxidant genes, promoting cardiolipin externalization for PINK1/Parkin-dependent mitophagy. Upon sustained stress, Nrf2/HO-1 depletion converts cardiolipin from an “eat-me” signal into a DAMP that directly activates NLRP3. (4) AMPK: Activated by metabolic stress, AMPK suppresses mTOR and promotes TFE3-driven expression of mitophagy-related genes via the SKP2-CARM1 axis. IRGM stabilizes p-AMPK to enhance ULK1/Beclin1 phosphorylation and autophagosome formation. Cross-talk: AMPK and NOD2-RIPK2 converge on ULK1 phosphorylation; HO-1-mediated cardiolipin externalization provides substrate for PINK1/Parkin. ROS is a shared upstream signal engaging both Nrf2/HO-1 (antioxidant) and NOD2-RIPK2 (inflammatory) pathways. All four pathways converge on NLRP3, whose activation via caspase-1/GSDMD executes pyroptosis.

#### 4.4.5 Comparative analysis of PINK1/Parkin and BNIP3/NIX versus receptor-mediated mitophagy in pyroptosis regulation

PINK1/Parkin is the most extensively characterized mitophagy pathway (27); however, receptor-mediated pathways, particularly BNIP3/NIX, also play crucial yet functionally distinct roles in regulating NLRP3-pyroptosis in various organs (30). The upstream triggers and downstream consequences of these pathways differ fundamentally, and these distinctions carry significant implications for tissue-specific therapeutic strategies.

PINK1/Parkin is highly sensitive to acute changes in MMP. When mitochondria depolarize, PINK1 accumulates on the outer mitochondrial membrane and recruits and activates Parkin, initiating ubiquitin-dependent mitophagy. This pathway operates mainly under acute stress conditions such as ischemia-reperfusion, toxin exposure, or sudden inflammatory insults (6, 112, 117, 124).

By contrast, BNIP3/NIX-mediated mitophagy is controlled mainly at the transcriptional level. BNIP3 is induced by HIF-1α during hypoxia, and NIX is regulated by FoxO3a and other transcription factors. This pathway therefore predominates under chronic hypoxia or metabolic stress, when sustained transcriptional reprogramming is required (40, 120, 135).

This functional divergence is evident across multiple models of organ injury. In kidney disease, Roxadustat, a hypoxia-inducible factor (HIF) inhibitor, enhances HIF-1α–BNIP3 signaling to restore BNIP3/NIX-mediated mitophagy, reduce mtROS, and suppress NLRP3 inflammasome activation, thereby attenuating tubular atrophy and fibrosis in adenine-induced chronic inflammation models. In contrast-induced acute kidney injury, Roxadustat likewise increases BNIP3 to reinstate mitophagy and decrease tubular cell death (135). Together, these findings indicate that activation of the BNIP3/NIX pathway—rather than PINK1/Parkin—is especially effective under hypoxia, when HIF-1α is stabilized (135).

In intervertebral disc (IVD) degeneration, A20 inhibits the mTOR/BNIP3 axis to promote BNIP3-driven mitophagy, which lowers mtROS release and prevents NLRP3 inflammasome assembly and pyroptosis in nucleus pulposus cells. This signaling cascade acts through the regulatory node mTOR and converges on BNIP3 independently of the PINK1/Parkin pathway (119, 120).

In postmenopausal osteoporosis (PMOP), estrogen deficiency impairs BNIP3-mediated mitophagy, leading to mtDNA release that activates the cGAS-STING-NLRP3 axis and triggers pyroptosis in bone marrow MSCs (136). This impairment contributes to an inflammation-dominated bone microenvironment and disrupts the balance between osteogenesis and adipogenesis. These findings further indicate that BNIP3 dysfunction can drive pyroptosis independently of alterations in the PINK1/Parkin pathway (136).

Importantly, these pathways are not mutually exclusive; they can display functional redundancy or cooperate depending on context. In acute alcoholic liver injury, both PINK1/Parkin- and BNIP3-mediated mitophagy activate as early protective responses, indicating that they can perform overlapping roles when rapid mitochondrial clearance is required (137). If PINK1/Parkin is impaired or overwhelmed, BNIP3/NIX can act as a compensatory backup, and the reverse may occur depending on the tissue and stress conditions.

From these observations, we propose a context-dependent model of pathway selection: PINK1/Parkin predominates under acute, MMP-driven stress, while BNIP3/NIX predominates under chronic hypoxia, metabolic stress, or conditions that involve transcriptional or epigenetic regulation of mitophagy receptors (40, 135, 136). The net effect on NLRP3-driven pyroptosis—protective or detrimental—reflects the integrity and sufficiency of the engaged pathway rather than the pathway identity itself (112, 124). This framework highlights the need for pathway-specific, rather than generalized, strategies when targeting mitophagy for therapeutic intervention across different organ diseases.

### 4.5 Temporal threshold and tissue-specific biomarkers

The idea that mitophagy functions within a “therapeutic window” is exemplified by the temporal pattern of CL externalization in HQ-treated retinal pigment epithelial cells. During the first 30 minutes to 2 hours of HQ exposure, a transient ROS burst induces Nrf2 nuclear translocation and HO-1 upregulation, which together promote CL externalization as an “eat-me” signal for PINK1/Parkin-dependent mitophagy and thereby facilitate removal of damaged mitochondria. In contrast, after prolonged HQ exposure (48 hours), sustained oxidative stress exhausts the Nrf2/HO-1 axis, and CL externalization shifts from a mitophagy-promoting cue to a damage-associated molecular pattern that directly activates the NLRP3 inflammasome, leading to caspase-1/GSDMD-dependent pyroptosis (98). This time-dependent switch shows that a single molecule can have protective or deleterious roles depending on the duration of the stress.

This temporal threshold is further limited by lysosomal degradative capacity. Excessive autophagic flux—as observed in high-fat diet-induced hepatic injury—overloads lysosomes, triggers LMP, and releases CTSB. Freed CTSB directly activates the NLRP3 inflammasome, shifting mitophagy from a protective process into a driver of pyroptosis apigenin (122). In Di (2-ethylhexyl) phthalate(DEHP)-induced testicular toxicity, the IRE1-XBP1 axis concurrently activates NLRP3/caspase-1-mediated pyroptosis and PINK1/Parkin-mediated mitophagy, implying that mitophagy is elicited as a compensatory protective response whose net effect depends on the relative kinetics of these opposing arms (138). In contrast-induced acute kidney injury (CI-AKI), mitophagy appears at 48 hours post-contrast, coincident with maximal Nlrp3 inflammasome activation and neutrophil infiltration, which raises whether this delayed response is “too little, too late” (35, 124). By contrast, in AngII-induced cardiomyocyte hypertrophy, hirudin simultaneously suppresses NLRP3 activation and enhances PINK1/Parkin-dependent mitophagy, providing pharmacological evidence that restoring balance—rather than simply activating or inhibiting a single pathway—is the optimal therapeutic strategy (139).

Tissue-specific differences in this threshold are evident. In the central nervous system, microglial pyroptosis predominantly depends on the canonical NLRP3/caspase-1/GSDMD axis, while neurons can undergo pyroptosis via caspase-3/GSDME or NLRP1 inflammasome–mediated routes (140, 141). This cell-type divergence has two important implications. First, within a single organ, the threshold at which mitophagy shifts from protective to pro-pyroptotic varies substantially between cell types—neurons are unusually prone to excessive mitophagy, which can disturb the caspase-3/GSDME balance and trigger pyroptosis, whereas microglia mainly activate the NLRP3 pathway (142). Second, therapeutic approaches must account for cellular heterogeneity, because broadly “enhancing mitophagy” in neurons may produce unintended pro-pyroptotic effects, as illustrated by PINK1 over-stabilization driving GSDME-dependent pyroptosis in postoperative cognitive dysfunction(POCD) models (142).

Based on these multi-organ observations, we propose the following candidate temporal biomarkers to distinguish protective from harmful mitophagy: (1) the duration of PINK1 accumulation on the outer mitochondrial membrane; (2) the kinetics of Parkin recruitment and completion of autophagic flux, assessed by LC3B-II turnover and p62 degradation; (3) the extent of CL externalization, whether transient or sustained; and (4) CTSB release from lysosomes, reflecting LMP and breach of a damage threshold. These biomarkers should be validated in clinical samples, and organ-specific reference ranges established for therapeutic monitoring.

## 5 Mechanism of pyroptosis and mitophagy interaction in organ damage

Research indicates a strong interconnection between pyroptosis and mitophagy in a network involving signals, metabolism, and inflammation. This bidirectional regulation occurs during organ injury and has been extensively studied in various organs. Tissue damage in these organs is often associated with abnormal mitochondrial function and an exacerbated inflammatory response, where pyroptosis and mitophagy play crucial roles. Targeted interventions at key points in this network can enable precise modulation to address organ damage effectively (**Figure 3** and **Table 1**).

**Figure 3:**
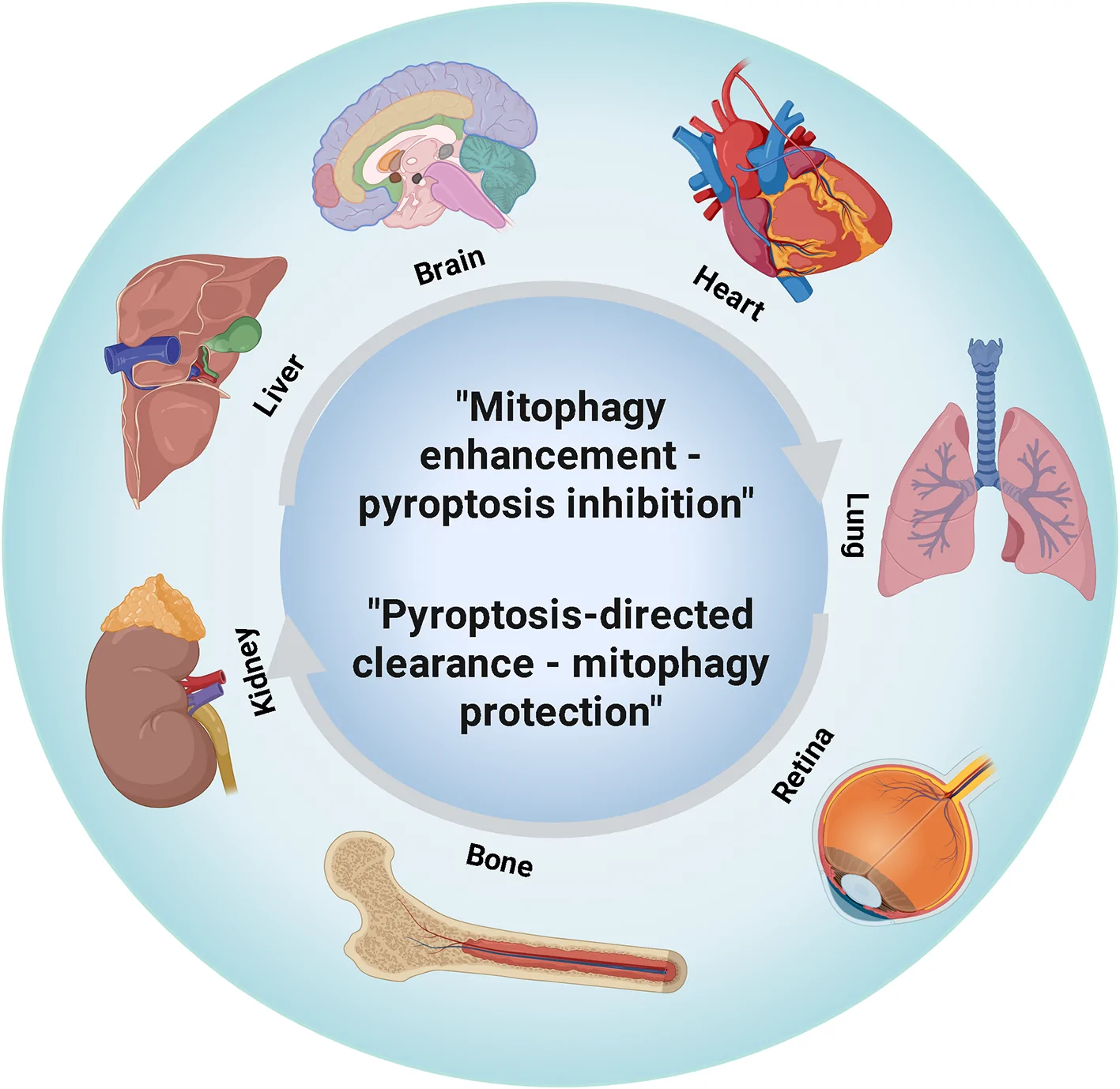
“Mitophagy enhancement - pyroptosis inhibition” refers to a protective strategy of inhibiting pyroptosis by enhancing mitophagy; “Pyroptosis - directed clearance - mitophagy protection” refers to a strategy of restoring the protective effect of mitophagy by directing the clearance of over - activated pyroptosis. Brain: Neuronal pyroptosis proceeds via caspase-3/GSDME or NLRP1 pathways, whereas microglial pyroptosis predominantly involves NLRP3/caspase-1/GSDMD. PINK1/Parkin-dependent mitophagy exerts neuroprotection. Liver: Deoxycholic acid inhibits PINK1-mediated mitophagy, and XBP1 deficiency impairs mitophagy, resulting in mtDNA–cGAS–STING–driven pyroptosis. Kidney: The OPTN/PINK1/Parkin axis and PHB2-facilitated Parkin translocation regulate NLRP3 activation, while roxadustat enhances BNIP3-mediated mitophagy. Lung: Crystalline silica induces mtROS–NLRP3 pyroptosis; ZBP1 senses mtDNA, and immunodeficiency compromises PINK1/Parkin mitophagy. Bone: BNIP3 promoter methylation in osteoporosis and the A20–mTOR–BNIP3 axis in disc degeneration modulate NLRP3-dependent pyroptosis. Heart: The SIRT6/PINK1 axis and the TXNIP–NLRP3 pathway regulate cardiomyocyte pyroptosis. Together, these spatiotemporal interactions enable a controlled switch between autophagy-mediated protection and pyroptotic clearance in injured organs.

**Table 1:** Mechanism of pyroptosis and mitophagy interaction in organ damage.

Organ	Disease model	Interaction mechanism	References
**Brain**	Hippocampal Oxidative Stress Model of Ischemia-Reperfusion Injury (HIR)	Dexmedetomidine → SIRT3↑ → PINK1/Parkin↑ → Mitophagy↑ → NLRP3↓ → GSDMD↓ → Pyroptosis↓ (Enhancing mitophagy via SIRT3 activation to suppress pyroptosis)	(143)
	Microglial Oxygen-Glucose Deprivation/Reoxygenation (OGD/R) Model	HucMSC-Ex → FOXO3a-dependent mitophagy↑ → GSDMD-N-terminal oligomerization↓ → pyroptosis↓ (Exosomes enhance autophagy via FOXO3a, suppressing microglial pyroptosis)	(144)
	Copper Exposure-Induced Parkinson’s Disease (PD) Model	Cu²^+^ accumulation → PINK1/Parkin↓ → mitophagy↓ → ROS↑ → NLRP3↑ → pyroptosis↑ (Copper ions inhibit autophagy initiation, leading to ROS burst and hyperactivation of the inflammatory axis)	(106)
	Cerebral Ischemia-Reperfusion (CIR) Model	Glycoside-based small molecules (e.g., G-Rg1) → PINK1↑-Parkin↑-mitophagy↑ → NLRP3-ASC-pro-caspase-1 macrocomplex↓ → pyroptosis↓ (Stabilizing the PINK1/Parkin axis to restore autophagic flux, dually suppressing inflammation and pyroptosis)	(145)
	Postoperative Cognitive Dysfunction (POCD)	Core Axis: PINK1/Parkin-mitophagy ↔ caspase-3/GSDME-pyroptosis Pathogenesis: Sustained high PINK1 expression → Release of caspase-3/GSDME inhibition → GSDME-N release → Rapid pyroptosis Protection: SNAP25↑ → PINK1 stabilization → mitophagy↑ → caspase-3/GSDME↓ → pyroptosis↓ Regulation: TNFAIP1 knockout → SNAP25 stabilization → Restoration of PINK1/Parkin axis → caspase-3↓ → pyroptosis↓	(140, 142, 146)
	Neuroinflammation Model	M@BPTLD → Mitochondrial targeting → Blockade of autophagosome-lysosome fusion → Accumulation of damaged mitochondria → GSDMD-N release → Dendritic cell activation → Systemic immune amplification (Utilizing the coupling of “excessive autophagy-pyroptosis” to artificially amplify immune signals)	(147)
	Autophagy Stimulation Model	Microglia: NLRP3/caspase-1/GSDMD axis; Neurons: caspase-3/GSDME or NLRP1 pathway (Different cell types employ distinct pyroptosis execution pathways)	(140, 141)
	Oxidative Stress-Pyroptosis Model (Transcutaneous Electrical Nerve Stimulation, TENS)	TENS → HIF-1α/BNIP3↑ + TXNIP/BRCC3-NLRP3↓ (Cooperatively activating protective autophagy pathways and inhibiting the inflammatory axis, dually suppressing oxidative stress and pyroptosis)	(148)
	General Protective Strategy for Ischemic Nerve Injury	Promoting mitophagy in ischemic neurons, clearing damaged mitochondria → NLRP3 inflammasome-mediated inflammatory response↓ → pyroptosis↓ → amelioration of brain injury (Clearance of damaged mitochondria is key to suppressing neuroinflammation and pyroptosis)	(149)
**Liver**	Nonalcoholic Steatohepatitis (NASH) Model	Deoxycholic acid → PINK1-mediated mitophagy↓ → NLRP3↑ → hepatocyte pyroptosis↑ (Inhibiting PINK1-dependent autophagy leads to inflammasome activation and pyroptosis)	(150–152)
	NASH-Related Liver Fibrosis (Intervention)	Liraglutide → Restoration of PINK1/Parkin pathway → Damaged mitochondria↓ → NLRP3 and downstream pyroptosis↓ (GLP-1RA restores mitochondrial quality control, inhibiting inflammation and fibrosis)	(153)
	Drug-Induced Liver Injury (ConA)	Ginsenoside Rb1 → PINK1/Parkin↑ → mitophagy↑ → ROS↓ → NLRP3↓ → pyroptosis↓ (Enhancing autophagy to clear ROS and suppress NLRP3)	(153)
	Liver Injury Induced by Subacute Rumen Acidosis (SARA)	SARA → Hepatic translocation of LPS → Disruption of mitochondrial homeostasis → NLRP3 activation → hepatocyte pyroptosis (Gut-derived LPS disrupts autophagic homeostasis and activates inflammation)	(154)
	Acute Alcoholic Liver Injury	PINK1/Parkin/BNIP3-mediated mitophagy ↑ (early protection) → Sustained injury → mtDNA leakage → Chronic inflammation	(137, 155–157)
	Acute Liver Injury (ALI)/XBP1-Deficiency Model	XBP1 deficiency → mitophagy↓ → ROS↑ → NLRP3/caspase-1/GSDMD↑ → pyroptosis↑ → mtDNA release → STING activation (Loss of a key ER stress factor inhibits autophagy, triggering pyroptosis and amplifying inflammation)	(158)
**Kidney**	Contrast-Induced Acute Kidney Injury (CI-AKI)(CI-AKI)	PINK1/Parkin deficiency → mtROS↑ → NLRP3/caspase-1↑ → renal tubular epithelial cell pyroptosis↑ (Autophagy deficiency amplifies oxidative stress and inflammation)	(124)
	CI-AKI/PHB2 Function Study	PHB2↓ → Impaired Parkin translocation → Mitochondrial damage → Sustained NLRP3 activation ↑ → Cell death↑ (Downregulation of PHB2 disrupts mitophagy initiation, leading to persistent inflammation)	(116)
	Diabetic Kidney Disease (DKD)	OPTN↑ → PINK1/Parkin-mitophagy↑ → mtDNA↓ → NLRP3↓ → Podocyte/renal tubular epithelial cell pyroptosis↓ (The autophagy receptor OPTN inhibits inflammation by enhancing mitophagy)	(115)
	Toxin-Induced Kidney Injury (e.g., 3-MCPD)	Toxin → PINK1/Parkin↓ → mtROS-mtDNA↑ → NLRP3-ASC-caspase-1↑ → GSDMD activation → Pyroptosis; Ginsenoside Rb1 (via TFEB) → mitophagy↑ → Blocks this pathway (The toxin inhibits the autophagy axis, triggering intense pyroptosis; Rb1 rescues autophagy via TFEB)	(159)
	Chronic Hypoxia-Hypoperfusion	HIF inhibitor Roxadustat → HIF-1α-BNIP3 signaling↑ → BNIP3/NIX-mitophagy↑ → mtROS↓ → NLRP3↓ → Renal function protection and fibrosis attenuation (Enhances protective autophagy by upregulating the HIF-1α-BNIP3 axis)	(135)
	Hyperuricemic Nephropathy	Urolithin A → mitophagy↑ → mtDNA leakage↓ → STING-NLRP3↓ → pyroptosis↓ → Fibrosis attenuation (Restores autophagy function and inhibits the STING-NLRP3 axis)	(160)
	Immune Microenvironment Regulation	FRC-Exos → PINK1/Parkin axis↑ → mitophagy↑ → NLRP3-ASC assembly↓ → GSDMD-mediated pyroptosis↓ → Renal function improvement (Exosomes deliver protective signals, activating the autophagy axis and inhibiting inflammation)	(161, 162)
**Lung**	Silicosis (Crystalline Silica (CS) Exposure)	CS → Mitochondrial damage → mtROS/mtDNA release → NLRP3↑ → Macrophage pyroptosis↑ → Fibrosis (positive feedback loop) (Particulate matter damages mitochondria, activating NLRP3 via DAMPs and driving fibrosis)	(163, 164)
	Sepsis-Associated Acute Lung Injury (S-ALI)	ZBP1 senses mtDNA → RIPK3/HIF-1α → mtROS↑ → NLRP3↑ → Alveolar epithelial cell pyroptosis↑ → Pulmonary edema (mtDNA sensing activates the RIPK3-mtROS-NLRP3 axis)	(165)
	Cardiac Arrest-Cardiopulmonary Resuscitation (CA/CPR) Model	Immunodeficiency → PINK1/Parkin↓ → Mitophagy↓ → mtROS↑ → NLRP3↑ → Alveolar macrophage pyroptosis↑; Urolithin A or PINK1 overexpression can restore. (Impaired autophagy in an immunodeficient context triggers a positive inflammatory feedback loop; autophagy inducers can reverse this)	(166, 167)
**Bones and Joints**	Intervertebral Disc Degeneration (IVD)	A20 → mTOR/BNIP3↓ → BNIP3-mitophagy↑ → Damaged mitochondria/mtROS↓ → NLRP3↓ → Nucleus pulposus cell pyroptosis↓ (A20 inhibits inflammation by enhancing BNIP3-mediated autophagy via mTOR suppression)	(119, 120)
	Accelerated Disc Degeneration Associated with Systemic Lupus Erythematosus (SLE)	KEAP1↑ → NRF2 degradation → NRF2/BNIP3/NIX-mitophagy↓ → mtDNA leakage → cGAS-STING-NLRP3↑ → Nucleus pulposus cell pyroptosis↑ (Dysregulation of the KEAP1/NRF2 axis impairs autophagy, activating the cGAS-STING inflammatory axis)	(168)
	LPS/ATP-Induced Osteoblast Injury	DDIT3/CHOP → PINK1/Parkin-mitophagy↓ → NLRP3↑ → Osteoblast pyroptosis↑ (A core endoplasmic reticulum stress factor inhibits autophagy and activates inflammation)	(169)
	Rheumatoid Arthritis (RA) Hypoxic Environment	Hypoxia → BNIP3-mitophagy↑ & NLRP3 activation; Mitophagy alleviates FLS pyroptosis by clearing ROS and suppressing HIF-1α/NLRP3 (Under hypoxia, BNIP3-mediated autophagy is a key protective mechanism inhibiting FLS pyroptosis)	(170)
	Osteoarthritis (OA)	p21↑ → NLRP3/caspase-1/ASC↑ → Chondrocyte pyroptosis↑ → Cell death↑ (The senescence-associated factor p21 promotes inflammasome activation and chondrocyte pyroptosis)	(171)
	Secondary Injury in Spinal Cord Injury (SCI)	Bexarotene → AMPK pathway → TFE3 nuclear translocation↑ → Mitophagy↑ → NLRP3↓ → Neuronal pyroptosis↓ → Motor function improvement (Enhances autophagy and inhibits neuronal pyroptosis via the AMPK-TFE3 axis)	(132)
	Postmenopausal Osteoporosis (PMOP)	Estrogen deficiency → BNIP3 methylation↑ → BNIP3-mitophagy↓ → mtDNA leakage → cGAS-STING-NLRP3↑ → BMSC pyroptosis↑; Demethylating agent 5-aza → BNIP3-mitophagy↑ → Pyroptosis↓ (Estrogen deficiency epigenetically suppresses BNIP3-mediated autophagy; the demethylating agent 5-aza restores BNIP3 expression and rescues autophagy)	(136)
**Heart**	Myocardial Ischemia-Reperfusion Injury (MIRI)	Sirt6-mitophagy↑ → NLRP3↓ → GSDMD↓ → Infarct size↓, Sirt6 exosomes → p62/Beclin-1↑ → AIM2/GSDMD↓ (Sirt6 and its exosomes inhibit pyroptosis through distinct mechanisms, protecting the myocardium)	(172)
	Diabetic Cardiomyopathy (DCM)	High glucose → AKT-FOXO1-SIRT3↓ → PINK1/Parkin-mitophagy↓ → mtROS↑ → Excessive NLRP3 activation (High glucose suppresses autophagy via the AKT-FOXO1-SIRT3 axis, leading to hyperactivation of inflammation)	(173)
	Septic Myocardial Injury	SIRT3 → Enhanced PINK1 deacetylation↑ → Mitophagy↑ → NLRP3-GSDMD↓ → Cardiomyocyte pyroptosis↓ (SIRT3 activates PINK1 through deacetylation, enhancing autophagy and inhibiting pyroptosis)	(174)
	Septic Myocardial Injury (HSP70 Protection)	HSP70 → Reversal of mitochondrial dysfunction → Inhibition of NLRP3 activation/GSDMD cleavage → Pyroptosis↓ → Improved cardiac function (The molecular chaperone helps restore mitochondrial function and directly inhibits the inflammatory axis)	(175)
	Environmental Toxin (Benzo[a]pyrene, BaP)-Induced Cardiac Injury	BaP → PINK1/Parkin-mPTP pathway → Excessive NLRP3 activation → Exacerbated post-myocardial infarction pyroptosis (Environmental toxins disrupt the PINK1/Parkin-mPTP axis, increasing pyroptosis risk after myocardial infarction)	(95)
	Chemotherapy-Induced Cardiotoxicity (Doxorubicin Model)	FSTL1 → Sirt6-Nrf2↑ → excessive autophagy/mitochondrial dysfunction↓ → Myocardial pyroptosis and fibrosis↓ (By upregulating the Sirt6-Nrf2 axis, it suppresses drug-induced excessive autophagy and secondary pyroptosis)	(176, 177)
**Retina**	Toxic Stimulation Model (Hydroquinone, HQ Treatment)	Core axis: Nrf2/HO-1 pathway activity → Determines the fate of cardiolipin (CL) externalization signals → Drives protective mitophagy or triggers inflammatory pyroptosis (This pathway is a key molecular switch determining whether cells progress toward repair or death.) Early Protective Phase (30 min): HQ → ROS↑ → Nrf2 nuclear translocation → ARE/antioxidant genes (HO-1, etc.)↑ → ROS↓, HO-1↑ → HO-1 products → PINK1/Parkin-dependent CL externalization → mitophagy↑ → Maintenance of ΔΨm, clearance of mtROS (Early stage: The Nrf2/HO-1 axis responds rapidly, Parkin recruitment → mitophagy↑.) Sustained Injury Phase (48 h): HQ (sustained) → Depletion of Nrf2/HO-1 axis → Shift in CL externalization signals → NLRP3 inflammasome assembly → Caspase-1 activation → GSDMD cleavage → Pyroptosis (Late stage: Caspase-1 engagement → pyroptosis↑)	(98)
	Diabetic Retinopathy	Core axis: Hyperglycemia → TXNIP↑ → (1) PINK1/Parkin-mitophagy↓ → Impaired lysosomal acidification; (2) TXNIP binds NLRP3 → Caspase-1 activation → Retinal cell pyroptosis↑ (forms a vicious cycle) (TXNIP acts as a dual switch disrupting autophagy and activating inflammation, forming a vicious cycle) Reverse Validation Axis: Auranofin → TrxR↓ → Trx oxidation↑ → RPE mitochondrial damage → Pyroptosis. (Inhibiting TrxR exacerbates oxidative stress, inversely confirming the TXNIP/Trx axis as a key driver) Targeted Therapy Axis: MCC950 → NLRP3↓ → Retinal leukocyte adhesion↓, vascular leakage↓ → Autophagic flux↑ (Specific inhibition of NLRP3 simultaneously reduces inflammation and vascular pathology while restoring autophagy)	(178–180)
**Others**	Preeclampsia (Placental Ischemia-Reperfusion)	PINK1↓ → Mitophagy↓ → mtROS↑ → NLRP3↑ → Trophoblast cell GSDMD-pyroptosis↑ → Amplification of maternal systemic inflammatory response (Impaired placental autophagy leads to mitochondrial stress, triggering trophoblast pyroptosis and amplifying maternal inflammation)	(181)
	DEHP Exposure (Phthalate, Adolescent)	DEHP → ERS → Mitophagy↑, Pyroptosis↑ → Germ cell death (DEHP upregulates both autophagy and pyroptosis via endoplasmic reticulum stress, leading to reproductive toxicity)	(138)
	Toxin DON Exposure (Carp Splenic Lymphocytes)	DON → Disruption of mitochondrial homeostasis → mtROS↑ → mtROS-NF-κB-NLRP3↑ → Lymphocyte pyroptosis↑ (The toxin disrupts mitochondrial homeostasis, activating the mtROS-NF-κB-NLRP3 axis)	(182)
	Vascular Endothelial Injury (TMAO)	TMAO → 1. Complex III disruption → PINK1/Parkin-mitophagy↑; 2. LPEAT regulation → Mitochondrial damage↑ → Mitophagy↑ → NAD^+^/Sirt3↓ → NLRP3↑ → Endothelial cell pyroptosis↑ (Gut metabolites disrupt endothelial mitochondrial homeostasis through dual pathways, ultimately activating NLRP3)	(183)
	Intestinal Ischemia-Reperfusion Injury	DEX → α2-AR-SIRT3 axis↑ → SIRT3: 1. Stabilizes PINK1 → Mitophagy↑; 2. HDAC3/p53↓ → Drp1 transcription↑ → mtROS-NLRP3↓ → Intestinal epithelial cell pyroptosis↓ (DEX exerts multi-target synergistic effects via SIRT3 to maintain mitochondrial network homeostasis and inhibit pyroptosis)	(184)
**Tumor**	Hepatocellular Carcinoma (HCC)	MLD → Mitochondrial damage (ΔΨm↓, TOM20↓, ROS↑) → Mitochondrial ROS-BNIP3 axis → PI3K/AKT/mTOR↓ + Caspase-3/NLRP3↑ → Concurrent pyroptosis and mitophagy → Amplified tumor suppression	(185)
	Colorectal Cancer (CRC)	Oxaliplatin + Bortezomib → JNK-Bcl-xL-Bax axis → Bax oligomerization, ΔΨm loss, cytochrome c release; Bcl-xL phosphorylation releases Beclin-1 → Autophagy initiation; Caspase-8 cleaves Beclin-1 → Autophagy suppression → Enhanced cell death	(186)

Bold text indicates different categories.

### 5.1 Mitophagy-pyroptosis in brain injury

The interplay between mitophagy and pyroptosis serves as a double-edged sword that influences the trajectory of neuroinflammation following brain injury. Recent evidence indicates that the dynamic equilibrium between these two processes, rather than the activation of a singular pathway, is crucial for neuronal survival and cognitive outcomes.

Dexmedetomidine enhances PINK1/Parkin-dependent Mitophagy by activating SIRT3 deacetylase, reducing NLRP3 inflammasome assembly and GSDMD cleavage, thus blocking IL-1β and IL-18 release (143). HucMSC-Ex promote Mitophagy via FOXO3a, reducing GSDMD oligomerization in microglia, inhibiting pyroptosis, and mitigating neuronal damage (144). In a Parkinson’s disease (PD) model induced by copper, disrupted PINK1/Parkin mitophagy due to Cu^2+^ accumulation leads to NLRP3/Caspase-1/GSDMD axis overactivation, causing inflammatory release and pyroptosis (106). Small glycoside molecules, facilitates Parkin recruitment, restores autophagic flux, weakens NLRP3-ASC-pro-caspase-1 complex formation, achieving “double inhibition” in the cerebral ischemia-reperfusion (CIR) model (145). Collectively, these findings indicate that moderate mitophagy upregulation can modulate NLRP3 inflammasomes, limiting pyroptosis propagation.

Mitophagy is widely considered essential for removing defective mitochondria, thereby preventing NLRP3 inflammasome assembly and maintaining cellular homeostasis. Recent evidence, however, indicates this relationship can be context-dependent; in some cases, mitophagy pathway dysregulation paradoxically promotes NLRP3 inflammasome formation and consequent pyroptosis. Excessive mitophagy can enhance pyroptosis signaling by inhibiting GSDME cleavage, leading to GSDME-N-terminal accumulation in the mitochondrial membrane. Restoration of caspase-3 activity releases GSDME-N-terminal, triggering pyroptosis and exacerbating POCD (142). M@BPTLD nanosystems exploit this mechanism by blocking autophagy-lysosomal fusion, causing damaged mitochondria accumulation and GSDMD-N release, activating dendritic cells to amplify neuroinflammation and systemic immunity (147).

Neurons can induce pyroptosis through non-classical pathways. Synaptosome-associated protein of 25 kDa (SNAP25) regulates PINK1 stability, extending its half-life, enhancing mitophagy flux, and inhibiting GSDME cleavage, thus preventing neuronal pyroptosis and improving POCD (140). Microglia and neurons respond differently to autophagy stimuli regarding pyroptosis. Neurons can undergo pyroptosis via caspase-3/GSDME or NLRP1 pathways (140, 141).TNFAIP1 knockout stabilizes SNAP25, activates PINK1/Parkin axis, reduces pyroptosis, and improves POCD (146) TENS activates autophagy and inhibits NLRP3 inflammasomes, reducing oxidative stress-pyroptosis (148). Prolonged inhibition of mitophagy reinforces ROS-NLRP3 loop, exacerbating brain injury. Promoting mitophagy in ischemic nerve cells inhibits NLRP3 inflammasomes, mitigating pyroptosis and brain damage (149).

### 5.2 Mitophagy-pyroptosis in liver injury

Nonalcoholic steatohepatitis (NASH) is a subtype of nonalcoholic fatty liver disease (NAFLD) characterized by lipid accumulation and involving mitochondrial dysfunction, inflammasome activation, and programmed cell death. Inflammasome activation can lead to caspase-1 activation, promoting liver fibrosis (150, 151). Deoxycholic acid has been found to activate NLRP3 inflammasomes by inhibiting PINK1-mediated mitophagy, inducing pyroptosis in hepatocytes. This imbalance in mitochondrial quality control is crucial in NASH progression (152).

GLP-1 receptor agonist liraglutide delays NASH-related liver fibrosis by restoring PINK1/Parkin pathway function, reducing damaged mitochondria accumulation, inhibiting NLRP3 inflammasome activation, and suppressing pyroptosis. Ginsenoside Rb1 (G-Rb1) enhances PINK1/Parkin-mediated mitophagy, decreases ROS production, inhibits NLRP3 inflammasome activation, and mitigates pyroptosis in hepatocytes (153). Subacute rumen acidosis disrupts mitophagy, activates NLRP3 inflammasomes, leading to hepatocyte pyroptosis and inflammatory damage. In acute alcoholic liver injury models, PINK1/Parkin and BNIP3-mediated mitophagy provide early protection (154). Prolonged impaired mitophagy can induce mtDNA leakage, leading to chronic inflammation and fibrosis (137, 155–157).

X-box binding protein 1 (XBP1) regulates ER stress in acute liver injury. Its absence inhibits mitophagy, raises ROS levels, activates NLRP3/caspase-1/GSDMD pathways, and triggers pyroptosis in hepatocytes. Pyroptosis releases mtDNA, intensifying inflammation by activating the STING pathway in macrophages (158).

Studies suggest a close interplay between mitophagy and pyroptosis in hepatocytes. On the one hand, impaired mitophagy allows damaged mitochondria to accumulate; the resulting burst of mtROS and release of mtDNA activate the NLRP3 inflammasome, driving pyroptotic cell death (158). Conversely, efficient clearance of dysfunctional mitochondria via the PINK1/Parkin pathway suppresses mtROS and NLRP3 inflammasome activation, thereby attenuating pyroptosis (187). Thus, mitophagy and pyroptosis form a mutually antagonistic axis in acute liver injury (188).

### 5.3 Mitophagy-pyroptosis in kidney injury

AKI and chronic kidney disease (CKD) frequently exhibit a triad involving mitochondrial quality control, inflammasome activation, and pyroptosis.

Deletion of PINK1 or Parkin in a model of contrast-induced acute kidney injury (CI-AKI) increases mtROS, NLRP3 inflammasome assembly, and caspase-1 activation, emphasizing the role of mitophagy defects in inflammation amplification. Conversely, enhancing PINK1/Parkin axis inhibits mtROS-NLRP3 cascade, reducing tubular cell death and histological damage. Downregulation of prohibitin-2 (PHB2) impedes Parkin translocation to mitochondria, activating NLRP3 inflammasomes and cell death (124). Overexpressing PHB2 restores mitochondrial homeostasis, inhibits NLRP3 complex formation, and reduces IL-1β and IL-18 release, alleviating AKI progression (116). In diabetic kidney disease (DKD) patients, low Optineurin (OPTN) expression correlates with high IL-1β and IL-18 levels. OPTN overexpression in podocytes restores PINK1/Parkin-mediated mitophagy, reducing mtDNA leakage, NLRP3 inflammasome activation, and pyroptosis in podocytes and tubular cells (115). These findings suggest that promoting mitophagy may inhibit NLRP3 inflammasome activation, reducing renal injury and inflammation.

Toxins like 3-monochloropropane-1,2-diol (3-MCPD) inhibit hinder the PINK1/Parkin axis, leading to mtROS-mtDNA accumulation, NLRP3-ASC-caspase-1 complex formation, GSDMD disruption, and pyroptosis in renal cells. G-Rb1 boosts mitophagy via TFEB, eliminating damaged mitochondria, blocking ROS-NLRP3 loop, reversing pyroptosis, and mitigating 3-MCPD-induced renal harm (115). These findings highlight the protective role of the mitophagy-NLRP3 axis.

Roxadustat, a HIF inhibitor, shows nephroprotective effects in chronic hypoxia-hypoperfusion scenarios. In adenine-induced chronic inflammation models, Roxadustat enhances HIF-1α-BNIP3 signaling, promotes BNIP3/NIX-mediated mitophagy, reduces mtROS, and suppresses NLRP3 inflammasome, lessening tubular atrophy and fibrosis. In acute CI-AKI models, Roxadustat boosts BNIP3, restores mitophagy, lowers tubular cell apoptosis, and enhances renal function (135). Urolithin A restores mitophagy, curbs mtDNA leakage, and inhibits STING-NLRP3 activation in hyperuric acid nephropathy, reducing NLRP3 inflammasome activation, IL-1β and IL-18 levels, and tubulointerstitial fibrosis (160).

CD5L-rich FRC-Exos target tubular epithelial cells to activate the PINK1/Parkin axis, enhance Mitophagy, block NLRP3-ASC inflammasome assembly, inhibit GSDMD-mediated pyroptosis, and reduce serum creatinine and urea nitrogen levels (161). In S-AKI, bone marrow MSCs enhance Parkin mitochondrial translocation via SIRT1 deacetylase, increase mitophagy flux, reduce mtDNA leakage and NLRP3 inflammasome activation, and decrease IL-1β/IL-18 levels and pyroptosis index in renal tissue. Zn^2+^ supplementation can further enhance these effects by inhibiting SIRT7 and increasing Parkin acetylation modifications (162).

### 5.4 Mitophagy-pyroptosis in lung injury

Lung injury can result from infections, exposure to harmful substances, trauma, and systemic inflammatory reactions. Chronic inflammation leading to fibrosis can cause tissue destruction, functional impairment, organ failure, and death. Silicosis, an interstitial pulmonary fibrosis disease caused by prolonged exposure to CS, involves a complex fibrosis mechanism. CS particles can harm the mitochondrial oxidative respiratory chain, leading to membrane potential depolarization, mitochondrial damage, release of mtROS and mtDNA as DAMPs, activation of NLRP3 inflammasomes, and induction of pyroptosis in macrophages, thus amplifying inflammation (163, 164). CS exposure triggers mitophagy and pyroptosis, establishing a feedback loop that regulates mitochondrial homeostasis and promotes silicosis fibrosis. Studies suggest that enhancing macrophage mitophagy can reduce mtROS bursts, prevent NLRP3 inflammasome activation and subsequent pyroptosis, and decrease CS-induced pulmonary fibrosis and lung function decline (112).

Z-DNA-binding protein 1 (ZBP1) in S-ALI detects mtDNA, triggering RIPK3-dependent mitochondrial damage. This process, in collaboration with HIF-1α, boosts glycolytic flux, leading to mtROS production. Subsequently, mtROS drive macrophage polarization towards the pro-inflammatory M1 phenotype, activate NLRP3 inflammasomes, induce pyroptosis, and cause pulmonary edema in alveolar epithelial cells. Disrupting ZBP1 or inhibiting RIPK3 can impede this pathway, improving alveolar-capillary barrier function (165).

In the non-obese diabetic/severe combined immunodeficient (NOD/SCID) mouse model of cardiac arrest (CA)/cardiopulmonary resuscitation (CPR), the immunodeficiency background suppressed PINK1 and Parkin expression, hindering mitophagy and causing accumulation of damaged mitochondria. This led to excessive mtROS release, activating NLRP3 inflammasomes, triggering pyroptosis in alveolar macrophages. The released IL-1β inhibited mitophagy, creating a “mtROS-NLRP3-pyroptosis” loop that amplifies inflammation. Overexpressing mitophagy inducers like urolithin A or PINK1 via adenoviral vectors restored mitochondrial clearance, disrupted the loop, reduced lung damage, myeloperoxidase (MPO) activity, and improved survival (166, 167).

### 5.5 Mitophagy-pyroptosis in bone injury

A20 inhibits natural plasmacytoid dendritic cell (NP cell) pyroptosis by regulating mitophagy and mitochondrial kinetic homeostasis through the mTOR/BNIP3 axis. This reduces p-mTOR levels, enhances BNIP3-mediated mitophagy, decreases mtROS release, and suppresses IL-1β and IL-18 secretion, thus preventing NLRP3 inflammasome assembly and delaying IVD degeneration (119, 120). In Systemic lupus erythematosus (SLE), KEAP1 overactivation leads to Nrf2 degradation, disrupting Nrf2/BNIP3/NIX mitophagy and activating the cGAS-STING-NLRP3 cascade, inducing NP cell pyroptosis and accelerating IVD degradation. Bardoxolone, an NRF2 activator, restores BNIP3-dependent mitophagy and mitigates IVD degeneration (168).

DNA damage-inducible transcript 3/C/EBP homologous protein (DDIT3/CHOP) inhibits PINK1/Parkin-mediated mitophagy and activates NLRP3 inflammasomes, promoting osteoblast pyroptosis (169). Both BNIP3-mediated mitophagy and NLRP3-mediated pyroptosis activate fibroblast-like synoviocyte (FLS) in rheumatoid arthritis under hypoxia. Mitophagy reduces ROS, inhibits HIF-1α/NLRP3, and mitigates FLS pyroptosis (170). In osteoarthritis (OA), chondrocyte pyroptosis is elevated, positively correlating with disease severity. Cyclin-dependent kinase inhibitor 1A (p21) upregulation in OA chondrocytes correlates with NLRP3, cleaved-caspase-1, and ASC levels, promoting pyroptosis (171). In SCI, the mitophagy-pyroptosis axis affects neurological prognosis. Bex activates AMPK-mTOR and AMPK-SKP2-CARM1, promoting TFE3 nuclear translocation, upregulating mitophagy genes, reducing ROS, inhibiting NLRP3-mediated pyroptosis, and enhancing motor function in SCI mice (132).

PMOP patients show vulnerable bone marrow mesenchymal stem cells (BMSCs) prone to pyroptosis. Estrogen deficiency elevates DNA methylation in BNIP3 promoter, hindering BNIP3-mediated Mitophagy. This obstruction triggers mtDNA release, activating cGAS-STING-NLRP3 pathway, inducing pyroptosis in BMSCs. 5-aza, a demethylating agent, can rectify BNIP3 expression, rebalancing BMSC pyroptosis and osteogenic-adipogenic differentiation (136).

### 5.6 Mitophagy-pyroptosis in heart injury

Mitochondrial dysfunction, impaired mitophagy, and NLRP3 inflammasome overactivation are closely associated with cardiac diseases like myocardial ischemia-reperfusion injury(MIRI) and diabetic cardiomyopathy (DCM). Mitophagy plays a crucial role in reducing pyroptosis and myocardial damage by eliminating damaged mitochondria, decreasing ROS production, and inhibiting NLRP3 inflammasome activation. Proper levels of mitophagy can control NLRP3 inflammasome activation, while inadequate or excessive autophagy can lead to uncontrolled cell death and worsened myocardial injury (189).

In MIRI, enhancing mitophagy through SIRT6 reduces GSDMD lysis, decreases the infarct area, and inhibits AIM2 inflammasome activation and GSDMD cleavage by upregulating p62 and Beclin-1 expression. This process reduces GSDMD cleavage and pyroptosis in endothelial cells, improving cardiac function, limiting the infarct area, and slowing atherosclerosis progression (172). In high glucose conditions, inhibiting the AKT-FOXO1-SIRT3 axis impairs PINK1/Parkin-dependent mitophagy, leading to damaged mitochondria accumulation, mtROS release, and excessive NLRP3 inflammasome activation, contributing to dysregulated pyroptosis in DCM (173). SIRT3 enhances PINK1 activity, promoting mitophagy and inhibiting NLRP3-GSDMD cascade, reducing pyroptosis in cardiomyocytes (174). Concurrently, heat shock protein 70 (HSP70) improves cardiac function by reversing mitochondrial dysfunction and inhibiting NLRP3 activation, GSDMD cleavage, and pyroptosis (175). Benzopyrene (BaP) overactivates NLRP3 inflammasomes, exacerbating pyroptosis post-myocardial infarction (95). Additionally, the chemotherapy drug folliclestatin-like 1 (FSTL1) inhibits doxorubicin-induced hyperautophagy and mitochondrial dysfunction, reducing myocardial pyroptosis and fibrosis (176, 177).

### 5.7 Mitophagy-pyroptosis in retinopathy

Redox imbalances are crucial in diabetic retinopathy (DR) development. Hyperglycemia increases thioredoxin (Trx) interacting protein (TXNIP) expression, hindering Trx function, leading to mitochondrial superoxide anion bursts. Prolonged oxidative stress activates mPTP, triggering excessive mitophagy via PINK1. In DR, hyperglycemia elevates TXNIP, inhibiting PINK1/Parkin-mediated mitophagy and lysosomal acidification, disrupting autophagic flux. TXNIP interacts with NLRP3, promoting inflammatory microbody formation and pyroptosis. Accumulated damaged mitochondria and ROS create a feedback loop, worsening DR progression. Studies show Auranofin mimics mitochondrial damage and pyroptosis by inhibiting TrxR, while MCC950 inhibits NLRP3 inflammasomes, reducing inflammation and restoring autophagy in a rat model of type 2 diabetes (178–180).

### 5.8 Mitophagy-pyroptosis in other organ injuries

Research indicates that in the placental ischemia-reperfusion model, reduced PINK1 expression in preeclampsia patients inhibits mitophagy, causing the accumulation of damaged mitochondria. This accumulation triggers mtROS release, activating NLRP3 inflammasomes, inducing GSDMD-mediated pyroptosis in trophoblast cells, and enhancing the maternal inflammatory response (181). Additionally, DEHP exposure induces pyroptosis in germ cells through endoplasmic reticulum stress, mitigated by mitophagy (138). Exposure to DON disrupts mitochondrial homeostasis in carp spleen lymphocytes, leading to increased mtROS levels, activation of the mtROS-NF-κ-dependent NLRP3 axis, and subsequent pyroptosis and inflammation (182).

In human umbilical vein endothelial cells (HUVECs), trimethylamine n-oxide (TMAO) exacerbates vascular endothelial damage through disrupting mitochondrial complex III, activating PINK1/Parkin-mediated mitophagy, and modulating lysophosphatidylethanolamine acyltransferase (LPEAT) to enhance phospholipid remodeling. Excessive Mitophagy depletes NAD+, reduces SIRT3 deacetylase activity, and triggers NLRP3 deacetylation, leading to endothelial pyroptosis and atherosclerotic plaque formation (183).In intestinal ischemia-reperfusion injury, DEX enhances SIRT3 deacetylase activity via the α2-adrenergic receptor-SIRT3 axis, promoting mitophagy and maintaining mitochondrial homeostasis. DEX also inhibits GSDMD-N membrane pore formation, reducing pyroptosis in intestinal epithelial cells and protecting intestinal barriers (184).

### 5.9 Mitophagy-pyroptosis in tumors

The regulatory interplay between mitophagy and pyroptosis in cancer is an emerging frontier, characterized by context-dependent and tumor-specific outcomes that vary with tumor type, genetic background, and therapeutic setting.

In hepatocellular carcinoma (HCC), Mallotucin D (MLD) damages mitochondria and activates the mitochondrial ROS–BNIP3 axis, which suppresses PI3K/AKT/mTOR signaling while activating caspase-3 and NLRP3; this sequence produces concurrent pyroptosis and mitophagy that amplify antitumor efficacy (185). In colorectal cancer (CRC), oxaliplatin combined with bortezomib engages the JNK–Bcl-xL–Bax axis to induce mitochondrial dysfunction and Beclin-1–mediated autophagy; subsequent caspase-8–mediated cleavage of Beclin-1 suppresses autophagy and thereby enhances cell death (186). The tumor-specific mitophagy–pyroptosis axis is context dependent: mitophagy can either suppress or enhance pyroptosis by modulating ROS. Zhu et al. (2025) showed that PINK1-mediated mitophagy limits GSDME-dependent pyroptosis in neuroblastoma, implying that late-stage autophagy/lysosomal inhibitors (e.g., chloroquine) might potentiate PINK1-targeting or ROS-generating therapies, although this possibility remains speculative (190).

## 6 Intervention strategies targeting the regulation of mitophagy and pyroptosis

### 6.1 Natural small molecules

Alliine: Alliine from garlic enhances PINK1/Parkin-mediated mitophagy and inhibits NLRP3 inflammasome-mediated pyroptosis in LPS-treated macrophages (121, 191).

Scoparone (6, 7-dimethoxycoumarin): Scoparone induces mitophagy by promoting LC3B colocalization with mitochondria, reducing mitochondrial damage and mtROS production, thus inhibiting NLRP3 inflammasome activation and the inflammatory response. It shows potential in treating NLRP3-related inflammatory conditions, improving survival rates in sepsis models, lowering IL-1β and TNF-α levels, and reducing inflammation in multiple organs. In an enteritis model, Scoparone alleviates weight loss, colon shortening, inflammatory cell infiltration, and bacterial load caused by C. rodentium infection (192).

Quercetin (Qu): Qu, a natural flavonoid, promotes mitophagy in microglia in a mouse model of Parkinson’s disease. This action enhances damaged mitochondria clearance, reduces mtROS accumulation, and decreases NLRP3 inflammasome activation. Consequently, neurodegeneration is alleviated, leading to improved motor function (193).

Apigenin: Apigenin, a natural flavonoid, inhibits NLRP3 inflammasome activation and hepatocyte pyroptosis by promoting mitophagy, reducing ROS production, stabilizing lysosomal membranes, and decreasing CTSB leakage. This alleviates liver damage caused by a high-fat diet (122).

Magnoflorine: An alkaloid from Magnolia plants inhibits NLRP3/caspase-1-mediated pyroptosis in CKD induced by high-fat and high-fructose diets. It enhances Parkin-PINK1-dependent mitophagy, restores mitochondrial function, and decreases tubular pyroptosis, leading to improved renal injury (194).

Bergapten (BeG): BeG, a natural polyphenol found in grapes and knotweed, exhibits antioxidant and anti-inflammatory properties. In an E. coli sepsis model, BeG mitigated inflammation in the lung, liver, and colon by inducing Mitophagy, suppressing ROS accumulation, and inhibiting NLRP3 inflammasome activation, IL-1β release, and GSDMD-mediated pyroptosis. In a Salmonella typhimurium (C. rodentium) intestinal infection model, BeG reduced weight loss, restored colon length, decreased bacterial load, and alleviated intestinal inflammation (195).

Honokiol: It is a natural biphenyl lignan derived from the traditional Chinese herb Magnolia officinalis. It inhibits the activation of NLRP3 inflammasomes by targeting the NF-kB/NLRP3 axis, activating the SIRT1/autophagy axis, and promoting autophagy, thereby improving severe lupus nephritis (196).

G-Rb1: Gs-Rb1, a triterpene saponin compound in traditional Chinese medicine like ginseng, American ginseng, and Panax notoginseng, exhibits biological activity. It reduces mtROS, inhibits NLRP3 inflammasomes, blocks pyroptosis, and mitigates acute liver injury induced by chloropropanol analogues and concanavalin a via PINK1/Parkin-mediated mitophagy activation (197).

Cryptotanshinone (CTS): CTS, a natural diterpene quinone from Lamiaceae plants, disrupts Ca²^+^ signaling, inhibits PINK1/Parkin-dependent mitophagy, and reduces mtROS. It interferes with NLRP3 inflammasome assembly and activation, mitigates pyroptosis, decreases caspase-1 activity and IL-1β secretion *in vivo* and *in vitro* (198).

Purple sweet potato anthocyanins: It is a class of water-soluble natural pigments extracted from the root of purple sweet potato. These anthocyanin flavonoid compounds promote Mitophagy via the Nrf2 signaling pathway and inhibit pyroptosis in alveolar macrophages, thereby providing a protective effect against lung inflammation caused by Klebsiella pneumoniae infection (199).

Dioscorea-derived allantoin: It is an allantoin component isolated from plants of the genus Dioscorea. It mitigates cyclophosphamide-induced premature ovarian failure in female rats by inhibiting apoptosis, reducing ΔΨm collapse, decreasing ROS generation, and suppressing both mitophagy and pyroptosis in ovarian granulosa cells (200).

Dioscin: It is a natural steroidal saponin that mitigates CS-induced lung inflammation and fibrosis by promoting macrophage mitophagy (201).

Acetyl zingerone: It derived from the natural product gingerone, enhances mitophagy via the PINK1/Parkin signaling pathway, thereby inhibiting chondrocyte pyroptosis and slowing the progression of OA (202).

Hirudin: It is a single-stranded small molecule polypeptide found in the salivary glands and secretions of medical leeches, promotes mitophagy by increasing mtDNA expression, activating the PINK1/Parkin pathway, down-regulating NLRP3 inflammasome-related cytokines, and reducing proteins associated with NLRP3 inflammasome activation, ultimately inhibiting Ang II-induced cardiomyocyte hypertrophy (139).

Iris combined with Dendrobium officinale: Iris combined with Dendrobium officinale promotes mitophagy, enhances mitochondrial quality control, inhibits NLRP3 inflammasome activation, decreases the release of inflammatory factors IL-18 and IL-1β, reduces cardiomyocyte pyroptosis, and improves myocardial injury in aged rats (203).

Active ingredients in whole rice bran: Extended exposure to cold enhances fatty acid transport and oxidation, triggering oxidative stress that prompts mitochondrial fusion and autophagy. This cascade results in mitochondrial damage, ROS release, NLRP3 inflammasome activation, and pyroptosis initiation. Whole rice bran supplementation counteracts these effects via antioxidants, reducing mitophagy and pyroptosis pathways. Its benefits include cardiac structure enhancement, metabolic regulation, oxidation control, and cell death inhibition (131).

HC067047 (TRPV4 inhibitor derived from small molecules): Activation of TRPV4 leads to Ca^2+^ influx, triggering CAMKIIα signaling, increasing oxidative stress, and mitophagy. Mitochondrial dysfunction releases DAMPs, activating NLRP3 inflammasomes, initiating pyroptosis. Inflammatory response from pyroptosis worsens mitochondrial damage and autophagy. The TRPV4 inhibitor HC067047 halts calcium influx-ROS axis, lessens mitochondrial damage and DAMPs release, breaks mitophagy-pyroptosis cycle, and restores colonic barrier integrity (204).

Tetherin modulators (small molecules of natural origin): Tetherin hinders LRPPRC from forming complexes with Beclin1 and Bcl-2, promoting Beclin1 release to bind to PI3KCIII, initiating autophagy. Autophagy inhibits tumorigenesis, boosts the immune response, and protects T cells from apoptosis and pyroptosis (205).

### 6.2 Traditional Chinese medicine compound and active ingredients

Glycosides of Buyang Huanwu decoction (BYHWD): BYHW glycosides inhibit NLRP3 inflammasome activation and pyroptosis by activating the Nrf2-mediated antioxidant signaling pathway and the PINK1/Parkin-mediated mitophagy, protecting neurons from oxidative stress and inflammatory damage, thus enhancing neurological function in mice with CIR (145, 206).

He’s Yang Chao Recipe (HSYC): HSYC protects ovarian cells from damage due to oxidative stress and inflammatory responses by inhibiting PINK1/Parkin-mediated mitophagy and NLRP3 inflammasome activation, which enhances ovarian function in POI mice (207).

15a (tanshinone IIA-sodium sulfonate-piperazine conjugate): Tanshinone IIA (TanllA), from danshen, has modest anti-inflammatory effects. Structural derivatives show enhanced anti-inflammatory properties. Compound 15a protects the kidneys by targeting the “mitophagy-mtROS-NLRP3-pyroptosis” pathway. It reduces mtROS, enhances mitophagy, suppresses NLRP3 inflammasome, and ameliorates renal damage in acute tubular necrosis (208).

MallotucinD (MLD): MLD from Croton crassifolius induces mitochondrial damage, reduces △ψm, down-regulates TOM20, increases intracellular ROS, inhibits the PI3K/AKT/mTOR pathway, and activates Caspase-3, NLRP3, and IL-1β release through the mitochondrial ROS-BNIP3 axis. These actions induce pyroptosis and Mitophagy, intensifying cell death signals and impeding tumor growth (185).

Xijiao Dihuang Decoction combined with Yinqiao Powder (XDY): XDY, based on traditional Chinese medicine, enhances autophagic flow, triggering mitochondrial phagocytosis to remove damaged mitochondria releasing mtROS. This reduces intracellular oxidative stress, prevents additional mitochondrial damage from ROS buildup, and regulates ROS/NLRP3 inflammasome/pyroptosis signaling during influenza infection (209).

Siwu Decoction: Siwu Decoction targets the hepatocyte-macrophage mtDNA metabolic axis to prevent *de novo* synthesis and release of damaged hepatocyte mtDNA, disrupting the TLR9-NF-κB loop and inhibiting M1 macrophage polarization. It also enhances PINK1/Parkin-mediated Mitophagy to reduce NLRP3 inflammasome formation and GSDMD-N-induced pyroptosis in hepatocytes, thus mitigating the panapoptosis-inflammatory cycle in NAFLD (**Table 2**) (210).

**Table 2:** Intervention strategies for targeted regulation of mitophagy and pyroptosis.

Category	Name	Source/composition	Primary mechanism of action	Representative disease/model	References
**Natural Small Molecule**	Alliin	Garlic	Alliin → PINK1/Parkin-mitophagy↑ → NLRP3-pyroptosis↓	LPS-treated macrophages	(121, 191)
	Scoparone (6,7-Dimethoxycoumarin)	Chinese herb Yin Chen Hao	Scoparone → Mitophagy↑, LC3B-mitochondrial co-localization↑ → Clearance of damaged mitochondria↑ & mtROS↓ → NLRP3 activation↓	Sepsis, enteritis models	(192)
	Quercetin (Qu)	Natural flavonoid	Qu → Microglial mitophagy↑ → Clearance of damaged mitochondria↑, mtROS↓ → NLRP3 activation↓ → Neurodegeneration↓	Parkinson’s disease mouse model	(193)
	Apigenin	Natural flavone compound	Apigenin → Mitophagy↑, ROS↓, lysosomal membrane stabilization (CTSB leakage↓) → NLRP3 inflammasome activation↓ → Hepatocyte pyroptosis↓	High-fat diet-induced liver injury	(122)
	Magnoflorine	Alkaloid from Magnoliaceae plants	Magnoflorine → PINK1/Parkin-mitophagy↑ → NLRP3/caspase-1-mediated pyroptosis↓ → Renal tubular injury↓	High-fat high-fructose-induced chronic kidney disease (CKD) model	(194)
	Resveratrol (Bergapten, BeG)	Grapes, Polygonum cuspidatum, etc. (Natural polyphenol)	BeG → Mitophagy↑ → Clearance of damaged mitochondria↑, ROS↓ → NLRP3 activation↓ → IL-1β/GSDMD-pyroptosis↓	E. coli sepsis, S. typhimurium intestinal infection models	(195)
	Honokiol	Chinese herb Hou Po (Biphenyl lignan)	Honokiol → NF-κB/NLRP3 axis↓, SIRT1/autophagy axis	Severe lupus nephritis	(196)
	Ginsenoside Rb1	Ginseng, American ginseng, Panax notoginseng (Triterpenoid saponin)	Gs-Rb1 → PINK1/Parkin-mitophagy↑ → mtROS↓, NLRP3↓ → Pyroptosis↓	Chloropropanol analog-induced renal cell pyroptosis; ConA-induced acute liver injury	(197)
	Cryptotanshinone (CTS)	Salvia miltiorrhiza (Diterpenoid quinone)	CTS → Ca²^+^ signaling↓ → PINK1/Parkin-mitophagy↓, mtROS↓ → Specific interference with NLRP3 assembly/activation → Pyroptosis↓	LPS or crystalline particle-induced lung injury and fibrosis	(198)
	Purple Sweet Potato Anthocyanin	Purple sweet potato (Anthocyanin flavonoid)	Anthocyanin → Nrf2 pathway↑ → Mitophagy↑ → Alveolar macrophage pyroptosis↓	Klebsiella pneumoniae (KP) infection-induced pulmonary inflammation	(199)
	Diosgenin Allantoin	Dioscorea plants	Diosgenin allantoin → Apoptosis↓, ΔΨm↓, ROS↓-mitophagy↓, pyroptosis↓	Cyclophosphamide-induced premature ovarian failure in female rats	(200)
	Dioscin	Natural steroidal saponin	Dioscin → Macrophage autophagy↑ → Inflammation and fibrosis↓	Silica dust-induced pulmonary inflammation and fibrosis	(201)
	Acetyl Zingerone	Derivative of natural zingerone	Acetyl zingerone → PINK1/Parkin signaling↑ → Mitophagy↑ → Chondrocyte pyroptosis↓	Osteoarthritis progression	(202)
	Hirudin	Medicinal leech salivary gland (Polypeptide)	Hirudin → mtDNA expression↑ → PINK1/Parkin signaling↑ → NLRP3 inflammasome-related factors↓	AngII-induced cardiomyocyte hypertrophy	(139)
	Irisin combined with Dendrobium candidum	Combination of irisin and Dendrobium candidum	Irisin + D. candidum → PINK1/Parkin pathway↑ → Mitophagy↑ → NLRP3 activation↓ & IL-18/IL-1β release↓ → Cardiomyocyte pyroptosis↓	Myocardial injury in aged rats	(203)
	Full-fat rice bran active components	Rice bran	Full-fat rice bran → Antioxidant↑ → Excessive mitophagy↓, pyroptosis pathway↓ → Cardiac injury↓	Long-term cold stress-induced cardiac injury	(131)
	HC067047	TRPV4 inhibitor (Naturally derived)	HC067047 → TRPV4↓ → Ca²^+^ influx-ROS axis↓ → Mitochondrial damage-DAMPs release↓ → NLRP3 activation↓ → Breaks the mitophagy-pyroptosis vicious cycle	Colonic barrier injury model	(204)
	Tetherin modulator	Naturally sourced small molecule	Tetherin modulator → Tetherin-LRPPRC interaction → Beclin1 release → Autophagy initiation↑ → Cell death (apoptosis/pyroptosis)↓	Tumor, immune-related models	(205)
**Traditional Chinese Medicine Formulations and Active Ingredients**	Bu Yang Huan Wu Decoction glycosides (BYHWD)	Astragalus, Angelica, etc. (Extract of Bu Yang Huan Wu Decoction)	BYHWD glycosides → Nrf2 pathway↑ → PINK1/Parkin-mitophagy↑ → NLRP3 inflammasome activation↓ → Pyroptosis↓	Cerebral ischemia-reperfusion injury	(145, 206)
	He Shi Yang Chao Formula (HSYC)	TCM formula (HSYC)	HSYC → PINK1/Parkin-mitophagy↓, NLRP3 inflammasome activation↓ → Ovarian cell protection	Premature ovarian insufficiency (POI) mouse model	(207)
	15a (Tanshinone IIA derivative)	Sodium tanshinone IIA sulfonate-piperazine conjugate	15a → Mitophagy↑, mtROS/mitochondrial damage↓ → NLRP3↓ → Pyroptosis↓	Acute tubular necrosis (ATN)	(208)
	Mallotucin D (MLD)	Chinese herb “Ji Gu Xiang” (Croton species, Clerodane diterpenoid)	MLD → Mitochondrial damage/ΔΨm↓/ROS↑ → Mitochondrial ROS-BNIP3 axis → PI3K/AKT/mTOR↓ → Caspase-3/NLRP3↑ → Mitophagy-pyroptosis ↑	Tumor model	(185)
	Xijiao Dihuang Decoction combined with Yinqiao Powder (XDY)	TCM-based formulation	XDY → Complete autophagic flux↑, mitophagy↑ → Clearance of damaged mitochondria releasing mtROS↑ → Intracellular ROS/oxidative stress↓ → Negative regulation of ROS/NLRP3/pyroptosis axis	Influenza virus infection model	(209)
	Si Wu Tang (Four Substances Decoction)	Rehmannia glutinosa (prepared), Angelica sinensis, Paeonia lactiflora, Ligusticum chuanxiong	Si Wu Tang → Hepatocyte mtDNA synthesis/release↓ → Blocks TLR9-NF-κB positive feedback → M1 macrophage polarization↓; Simultaneously PINK1/Parkin-mitophagy flux↑ → NLRP3/GSDMD-N-mediated hepatocyte pyroptosis↓	Non-alcoholic fatty liver disease (NAFLD)	(210)

Bold text indicates different categories.

### 6.3 Translational barriers and perspectives

Despite strong preclinical evidence that natural small molecules and TCM formulations can modulate the mitophagy–pyroptosis axis, several translational hurdles remain. First, many of these compounds have poor oral bioavailability and rapid metabolism. For example, quercetin and apigenin are extensively glucuronidated and sulfated in the intestine and liver, yielding plasma concentrations well below those needed for *in vitro* effects. Second, their organ distribution is largely nonspecific, which raises concerns about off-target effects and about achieving therapeutic levels in privileged sites such as the brain (because of the blood–brain barrier) or the kidney. Third, *in vivo* pharmacokinetic (PK) data are lacking for most listed agents, and most studies use intraperitoneal or intravenous dosing in rodents—routes that do not mimic clinical administration. Fourth, to our knowledge no clinical trial has specifically tested any of these compounds as modulators of the mitophagy–pyroptosis axis; evidence remains limited to cell lines and rodent models. These limitations do not negate the value of these natural products as leads but underscore the urgent need for medicinal chemistry optimization (for example, prodrug strategies or nanoparticle encapsulation), rigorous PK/PD characterization, and well designed phase I/II clinical trials to translate these promising preclinical findings into clinical practice (211, 212).

## 7 Discussion and outlook

This study explores the interaction between pyroptosis and mitophagy in different diseases. Pyroptosis is a pro-inflammatory cell death process involving gasdermin proteins, while mitophagy selectively removes damaged mitochondria to maintain cellular balance. Recent research reveals a complex relationship between these processes, impacting disease development. Mitophagy regulates pyroptosis by reducing mtROS and mtDNA through damaged mitochondria removal, thus managing cellular energy and inflammation. Conversely, pyroptosis can hinder mitophagy, forming a negative feedback loop. The discussion focuses on their interplay in tissue injuries, evident in brain, liver, kidney, lung, bone, and heart conditions, showcasing diverse regulatory mechanisms. The precise balance between the pyroptosis and mitophagy pathways is crucial for the host’s ability to resist inflammatory damage and maintain homeostasis. Disruption of balance between pyroptosis and mitophagy can lead to a harmful cycle of inflammation and mitochondrial damage. While some interactions between these pathways are known, gaps persist in understanding their molecular network. Key questions involve identifying specific molecules regulating this balance, the effects on cell function, and the role of different mitophagy pathways in pyroptosis. Investigating potential redundancy between pathways and improving detection methods are crucial for developing effective anti-pyroptosis treatments targeting mitophagy.

Our review revealed a paradox: Mitophagy dysregulation can paradoxically induce NLRP3 inflammasome activation and pyroptosis through two imbalanced states. Insufficient mitophagy leads to the accumulation of damaged mitochondria, triggering NLRP3 activation and pyroptosis. Excessive mitophagy results in autophagic overload, preventing complete clearance of defective mitochondria and leading to NLRP3 activation and pyroptosis. Maintaining moderate mitophagic activity is crucial for preventing inflammatory cell death. Therapeutic approaches should aim to restore mitophagic activity to physiological levels to interrupt the inflammatory cycle. IL-1β released during pyroptosis can induce mitophagy as a compensatory response to remove damaged mitochondria and limit persistent inflammation. The specific molecular mechanisms through which IL-1β triggers mitophagy are not fully understood and require further investigation. Identifying and studying the components of this feedback loop over different disease stages is crucial. IL-1β not only functions as an inflammatory mediator but also regulates dynamic responses. Targeting this loop, such as enhancing protective autophagy in early disease or inhibiting inflammation in chronic stages, could offer new strategies for immune modulation.

Future breakthrough pathways may encompass the following approaches: (1) coupling chemogenetics with CRISPRa/i to construct an inducible model for “Parkin amplification” or “GSDMD inhibition”, thereby enabling bidirectional intervention at the single-cell level. (2) employing multimodal imaging, where the △ψm-ROS-gasdermin three-channel near-infrared probe to create a live, real-time “mitophagy -pyroptosis” scorecard. (3) Utilizing AI-assisted target mining, which integrates single-cell transcriptome analysis with protein interaction networks to predict bifunctional small molecules capable of “simultaneously inhibiting NLRP3 and activating Parkin”, thus reducing the drug discovery cycle. (4) Develop tissue-specific delivery platforms to translate mitophagy–pyroptosis modulation into clinical practice. Nanoparticle-based systems (for example, lipid nanoparticles, exosomes, and peptide-modified carriers) have shown proof-of-concept efficacy in preclinical models, but clinical translation faces major obstacles, including limited pharmacokinetic data, unclear immunogenicity, and a lack of large-animal validation. Future work should prioritize optimization of delivery modalities alongside mechanistic studies to achieve targeted, safe, and effective modulation of this axis in specific organs.

Mitophagy is essential for cell survival and homeostasis, while pyroptosis serves dual roles: under physiological conditions, it eliminates intracellular pathogens, but when overactivated, it contributes to the pathological progression of various inflammatory and degenerative diseases. Therefore, a comprehensive understanding of the interplay between pyroptosis and mitophagy, as well as the specific mechanisms underlying pyroptosis, is vital for advancing human health and addressing disease development. Through interdisciplinary research, significant breakthroughs in this area are anticipated to offer new theoretical insights and practical guidance for enhancing human health and improving disease treatment.

## References

[1] West AP Shadel GS Ghosh S . Mitochondria in innate immune responses. Nat Rev Immunol. (2011) 11:389–402. doi: 10.1038/nri2975 21597473 PMC4281487

[2] Weinberg SE Sena LA Chandel NS . Mitochondria in the regulation of innate and adaptive immunity. Immunity. (2015) 42:406–17. doi: 10.1016/j.immuni.2015.02.002 25786173 PMC4365295

[3] Gurung P Lukens JR Kanneganti T-D . Mitochondria: diversity in the regulation of the NLRP3 inflammasome. Trends Mol Med. (2015) 21:193–201. doi: 10.1016/j.molmed.2014.11.008 25500014 PMC4352396

[4] Kasahara A Scorrano L . Mitochondria: from cell death executioners to regulators of cell differentiation. Trends Cell Biol. (2014) 24:761–70. doi: 10.1016/j.tcb.2014.08.005 25189346

[5] Youle RJ Narendra DP . Mechanisms of mitophagy. Nat Rev Mol Cell Biol. (2011) 12:9–14. doi: 10.1038/nrm3028 PMC4780047 21179058

[6] Eiyama A Okamoto K . PINK1/Parkin-mediated mitophagy in mammalian cells. Curr Opin Cell Biol. (2015) 33:95–101. doi: 10.1016/j.ceb.2015.01.002 25697963

[7] A.A MA Ameenudeen S Kumar A Hemalatha S Ahmed N Ali N . Emerging role of mitophagy in inflammatory diseases: cellular and molecular episodes. Curr Pharm Des. (2020) 26:485–91. doi: 10.2174/1381612826666200107144810 31914907

[8] Zhao Y Huang S Liu J Wu X Zhou S Dai K . Mitophagy contributes to the pathogenesis of inflammatory diseases. Inflammation. (2018) 41:1590–600. doi: 10.1007/s10753-018-0835-2 29959626

[9] Casey AM Ryan DG Prag HA Chowdhury SR Marques E Turner K . Pro-inflammatory macrophages produce mitochondria-derived superoxide by reverse electron transport at complex I that regulates IL-1β release during NLRP3 inflammasome activation. Nat Metab. (2025) 7:493–507. doi: 10.1038/s42255-025-01224-x 39972217 PMC11946910

[10] Chu T Huang Z Ma W . Mitophagy: a double-edged sword in tumor cell death regulation and therapeutic response. Biochem Biophys Res Commun. (2025) 777:152254. doi: 10.1016/j.bbrc.2025.152254 40578284

[11] Wang J Toan S Zhou H . Mitochondrial quality control in cardiac microvascular ischemia-reperfusion injury: new insights into the mechanisms and therapeutic potentials. Pharmacol Res. (2020) 156:104771. doi: 10.1016/j.phrs.2020.104771 32234339

[12] Friedlander AM . Macrophages are sensitive to anthrax lethal toxin through an acid-dependent process. J Biol Chem. (1986) 261:7123–6. doi: 10.1016/s0021-9258(17)38364-3 3711080

[13] Cookson BT Brennan MA . Pro-inflammatory programmed cell death. Trends in microbiology. 9(3):113–114. doi: 10.1016/s0966-842x(00)01936-3 11303500

[14] Martinon F Burns K Tschopp J . The inflammasome: a molecular platform triggering activation of inflammatory caspases and processing of proIL-beta. Mol Cell. (2002) 10:417–26. doi: 10.1016/S1097-2765(02)00599-3 12191486

[15] Yang J Jiang J . Gasdermins: a dual role in pyroptosis and tumor immunity. Front Immunol. (2024) 15. doi: 10.3389/fimmu.2024.1322468 38304430 PMC10830654

[16] Yang M Wei X Yi X Jiang DS . Mitophagy-related regulated cell death: molecular mechanisms and disease implications. Cell Death Dis. (2024) 15. doi: 10.1038/s41419-024-06804-5 39013891 PMC11252137

[17] Ashrafi G Schwarz TL . The pathways of mitophagy for quality control and clearance of mitochondria. Cell Death Differ. (2013) 20:31–42. doi: 10.1038/cdd.2012.81 PMC3524633 22743996

[18] Yoo SM Jung YK . A molecular approach to mitophagy and mitochondrial dynamics. Cells. (2018). 10.14348/molcells.2018.2277 PMC5792708 29370689

[19] Lemasters JJ . Selective mitochondrial autophagy, or mitophagy, as a targeted defense against oxidative stress, mitochondrial dysfunction, and aging. (2005). 10.1089/rej.2005.8.3 15798367

[20] Bingol B Sheng M . Mechanisms of mitophagy: PINK1, Parkin, USP30 and beyond. Free Radical Biol Med. (2016) 100:210–22. doi: 10.1016/j.freeradbiomed.2016.04.015 27094585

[21] Suomalainen A Nunnari J . Mitochondria at the crossroads of health and disease. Cell. (2024) 187:2601–27. doi: 10.1016/j.cell.2024.04.037 38788685

[22] Palikaras K Lionaki E Tavernarakis N . Balancing mitochondrial biogenesis and mitophagy to maintain energy metabolism homeostasis. Cell Death Differ. (2015) 22:1399–401. doi: 10.1038/cdd.2015.86 26256515 PMC4532782

[23] Li A Gao M Liu B Qin Y Chen L Liu H . Mitochondrial autophagy: molecular mechanisms and implications for cardiovascular disease. Cell Death Dis. (2022) 13. doi: 10.1038/s41419-022-04906-6 35534453 PMC9085840

[24] Chen Y Li Z Zhang H Chen H Hao J Liu H . Mitochondrial metabolism and targeted treatment strategies in ischemic-induced acute kidney injury. Cell Death Discov. (2024) 10. doi: 10.1038/s41420-024-01843-5 38341438 PMC10858869

[25] Lin C Chao H Li Z Xu X Liu Y Hou L . Melatonin attenuates traumatic brain injury‐induced inflammation: a possible role for mitophagy. J Pineal Res. (2016) 61:177–86. doi: 10.1111/jpi.12337 27117839

[26] Levine B Kroemer G . Biological functions of autophagy genes: a disease perspective. Cell. (2019) 176:11–42. doi: 10.1016/j.cell.2018.09.048 30633901 PMC6347410

[27] Nguyen TN Padman BS Lazarou M . Deciphering the molecular signals of PINK1/Parkin mitophagy. Trends Cell Biol. (2016) 26:733–44. doi: 10.1016/j.tcb.2016.05.008 27291334

[28] Um J-H Yun J . Emerging role of mitophagy in human diseases and physiology. BMB Rep. (2017) 50:299–307. doi: 10.5483/bmbrep.2017.50.6.056 28366191 PMC5498140

[29] Pickrell AM Youle RJ . The roles of PINK1, Parkin, and mitochondrial fidelity in Parkinson’s disease. Neuron. (2015) 85:257–73. 10.1016/j.neuron.2014.12.007 PMC4764997 25611507

[30] Sun K Jing X Guo J Yao X Guo F . Mitophagy in degenerative joint diseases. Autophagy. (2021) 17:2082–92. doi: 10.1080/15548627.2020.1822097 PMC8496714 32967533

[31] Okamoto K . Organellophagy: eliminating cellular building blocks via selective autophagy. J Cell Biol. (2014) 205:435–45. doi: 10.1083/jcb.201402054 24862571 PMC4033777

[32] Aoki Y Kanki T Hirota Y Kurihara Y Saigusa T Uchiumi T . Phosphorylation of serine 114 on Atg32 mediates mitophagy. Mol Biol Cell. (2011) 22:3206–17. doi: 10.1091/mbc.e11-02-0145 21757540 PMC3164466

[33] Furukawa K Fukuda T Yamashita SI Saigusa T Kurihara Y Yoshida Y . The PP2A-like protein phosphatase Ppg1 and the Far complex cooperatively counteract CK2-mediated phosphorylation of Atg32 to inhibit mitophagy. Cell Rep. (2018) 23:3579–90. doi: 10.1016/j.celrep.2018.05.064 29925000

[34] Sakakibara K Eiyama A Suzuki SW Sakoh-Nakatogawa M Okumura N Tani M . Phospholipid methylation controls Atg32‐mediated mitophagy and Atg8 recycling. EMBO J. (2015) 34:2703–19. doi: 10.15252/embj.201591440 26438722 PMC4641534

[35] Onishi M Yamano K Sato M Matsuda N Okamoto K . Molecular mechanisms and physiological functions of mitophagy. EMBO J. (2021) 40. doi: 10.15252/embj.2020104705 33438778 PMC7849173

[36] Richter B Sliter DA Herhaus L Stolz A Wang C Beli P . Phosphorylation of OPTN by TBK1 enhances its binding to Ub chains and promotes selective autophagy of damaged mitochondria. Proc Natl Acad Sci. (2016) 113:4039–44. doi: 10.1073/pnas.1523926113 27035970 PMC4839414

[37] Bingol B Tea JS Phu L Reichelt M Bakalarski CE Song Q . The mitochondrial deubiquitinase USP30 opposes parkin-mediated mitophagy. Nature. (2014) 510:370–5. doi: 10.1038/nature13418 24896179

[38] Cornelissen T Vilain S Vints K Gounko N Verstreken P Vandenberghe W . Deficiency of parkin and PINK1 impairs age-dependent mitophagy in Drosophila. eLife. (2018) 7. doi: 10.7554/elife.35878 29809156 PMC6008047

[39] Liang JR Martinez A Lane JD Mayor U Clague MJ Urbé S . USP 30 deubiquitylates mitochondrial P arkin substrates and restricts apoptotic cell death. EMBO Rep. (2015) 16:618–27. doi: 10.15252/embr.201439820 25739811 PMC4428036

[40] Madhu V Boneski PK Silagi E Qiu Y Kurland I Guntur AR . Hypoxic regulation of mitochondrial metabolism and mitophagy in nucleus pulposus cells is dependent on HIF-1α–BNIP3 axis. J Bone Miner Res. (2020) 35:1504–24. doi: 10.1002/jbmr.4019 32251541 PMC7778522

[41] Xu W Che Y Zhang Q Huang H Ding C Wang Y . Apaf-1 pyroptosome senses mitochondrial permeability transition. Cell Metab. (2021) 33:424–436.e10. doi: 10.1016/j.cmet.2020.11.018 33308446

[42] Kadono K Kageyama S Nakamura K Hirao H Ito T Kojima H . Myeloid Ikaros–SIRT1 signaling axis regulates hepatic inflammation and pyroptosis in ischemia-stressed mouse and human liver. J Hepatol. (2022) 76:896–909. doi: 10.1016/j.jhep.2021.11.026 34871625 PMC9704689

[43] Koh EH Yoon JE Ko MS Leem J Yun JY Hong CH . Sphingomyelin synthase 1 mediates hepatocyte pyroptosis to trigger non-alcoholic steatohepatitis. Gut. (2021) 70:1954–64. doi: 10.1136/gutjnl-2020-322509 PMC8458090 33208407

[44] Broz P . Pyroptosis: molecular mechanisms and roles in disease. Cell Res. (2025) 35:334–44. doi: 10.1038/s41422-025-01107-6 40181184 PMC12012027

[45] Schroder K Tschopp J . The inflammasomes. Cell. (2010) 140:821–32. doi: 10.1016/j.cell.2010.01.040 20303873

[46] Wree A Eguchi A McGeough MD Pena CA Johnson CD Canbay A . NLRP3 inflammasome activation results in hepatocyte pyroptosis, liver inflammation, and fibrosis in mice. Hepatology. (2014) 59:898–910. doi: 10.1002/hep.26592 23813842 PMC4008151

[47] Zychlinsky A Prevost MC Sansonetti PJ . Shigella flexneri induces apoptosis in infected macrophages. Nature. (1992) 358:167–9. doi: 10.1038/358167a0 1614548

[48] Weinrauch Y Zychlinsky A . The induction of apoptosis by bacterial pathogens. Annu Rev Microbiol. (1999) 53:155–87. doi: 10.1146/annurev.micro.53.1.155 10547689

[49] Ding J Wang K Liu W She Y Sun Q Shi J . Pore-forming activity and structural autoinhibition of the gasdermin family. Nature. (2016) 535:111–6. doi: 10.1038/nature18590 27281216

[50] Feng S Fox D Man SM . Mechanisms of gasdermin family members in inflammasome signaling and cell death. J Mol Biol. (2018) 430:3068–80. doi: 10.1016/j.jmb.2018.07.002 29990470

[51] Jorgensen I Rayamajhi M Miao EA . Programmed cell death as a defence against infection. Nat Rev Immunol. (2017) 17:151–64. doi: 10.1038/nri.2016.147 28138137 PMC5328506

[52] Kayagaki N Stowe IB Lee BL O'Rourke K Anderson K Warming S . Caspase-11 cleaves gasdermin D for non-canonical inflammasome signalling. Nature. (2015) 526:666–71. doi: 10.1038/nature15541 26375259

[53] Wang Y Gao W Shi X Ding J Liu W He H . Chemotherapy drugs induce pyroptosis through caspase-3 cleavage of a gasdermin. Nature. (2017) 547:99–103. doi: 10.1038/nature22393 28459430

[54] Zhou Z He H Wang K Shi X Wang Y Su Y . Granzyme A from cytotoxic lymphocytes cleaves GSDMB to trigger pyroptosis in target cells. Science. (2020) 368:eaaz7548. doi: 10.1126/science.aaz7548 32299851

[55] Hou J Zhao R Xia W Chang CW You Y Hsu JM . PD-L1-mediated gasdermin C expression switches apoptosis to pyroptosis in cancer cells and facilitates tumour necrosis. Nat Cell Biol. (2020) 22:1264–75. doi: 10.1038/s41556-020-0575-z 32929201 PMC7653546

[56] Liu X Zhang Z Ruan J Pan Y Magupalli VG Wu H . Inflammasome-activated gasdermin D causes pyroptosis by forming membrane pores. Nature. (2016) 535:153–8. doi: 10.1038/nature18629 27383986 PMC5539988

[57] Wang K Sun Q Zhong X Zeng M Zeng H Shi X . Structural mechanism for GSDMD targeting by autoprocessed caspases in pyroptosis. Cell. (2020) 180:941–955.e20. doi: 10.1016/j.cell.2020.02.002 32109412

[58] Zhang L Hu Z Li Z Lin Y . Crosstalk among mitophagy, pyroptosis, ferroptosis, and necroptosis in central nervous system injuries. Neural Regener Res. (2024) 19:1660–70. doi: 10.4103/1673-5374.389361 PMC10960298 38103229

[59] Shi J Zhao Y Wang Y Gao W Ding J Li P . Inflammatory caspases are innate immune receptors for intracellular LPS. Nature. (2014) 514:187–92. doi: 10.1038/nature13683 25119034

[60] Yuan J Najafov A Py BF . Roles of caspases in necrotic cell death. Cell. (2016) 167:1693–704. doi: 10.1016/j.cell.2016.11.047 27984721 PMC5381727

[61] Yang D He Y Muñoz-Planillo R Liu Q Núñez G . Caspase-11 requires the Pannexin-1 channel and the purinergic P2X7 pore to mediate pyroptosis and endotoxic shock. Immunity. (2015) 43:923–32. doi: 10.1016/j.immuni.2015.10.009 26572062 PMC4795157

[62] Dubey SR Turnbull C Pandey A Zhao A Kurera M Al-Zidan R . Molecular mechanisms and regulation of inflammasome activation and signaling: sensing of pathogens and damage molecular patterns. Cell Mol Immunol. (2025) 22:1313–44. doi: 10.1038/s41423-025-01354-y 41062723 PMC12575685

[63] Di Virgilio F Dal Ben D Sarti AC Giuliani AL Falzoni S . The P2X7 receptor in infection and inflammation. Immunity. (2017) 47:15–31. doi: 10.1016/j.immuni.2017.06.020 28723547

[64] Tong X Tong Y Zheng J Shi R Liang H Li M . TRPM7 contributes to pyroptosis and its involvement in status epilepticus. J Neuroinflamm. (2024) 21. doi: 10.1186/s12974-024-03292-4 39617893 PMC11608501

[65] Mohseni G Li J Ariston Gabriel AN Du L Wang YS Wang C . The function of cGAS-STING pathway in treatment of pancreatic cancer. Front Immunol. (2021) 12. doi: 10.3389/fimmu.2021.781032 34858438 PMC8630697

[66] Li H Wang X Luo X Shi H Li J . cGAS/STING/NLRP3 signaling pathway-mediated pyroptosis in hypertrophic cardiomyopathy radiotherapy. Front Bioscience-Landmark. (2025) 30. doi: 10.31083/fbl26084 40152382

[67] Zhang T Zou Y Zhang SH Wang YY He S Yuan W . Taletrectinib promotes pyroptosis in colorectal carcinoma via SRC/AKT/mTOR axis inhibition. Sci Rep. (2025) 15. doi: 10.1038/s41598-025-02901-3 40410374 PMC12102285

[68] Yu D Wang S Wang J Zhang K Niu Z Lin N . EZH2–STAT3 signaling pathway regulates GSDMD-mediated pyroptosis in glioblastoma. Cell Death Discov. (2024) 10. doi: 10.1038/s41420-024-02105-0 39069522 PMC11284224

[69] Wang J Wu Z Zhu M Zhao Y Xie J . ROS induced pyroptosis in inflammatory disease and cancer. Front Immunol. (2024) 15. doi: 10.3389/fimmu.2024.1378990 39011036 PMC11246884

[70] Ma L Han Y Chen Z Liu X Wang Y Wang Z . Transition of cellular senescence to pyroptosis mediates recurrence of small cell lung cancer after chemotherapy. Sci Adv. (2025) 11. doi: 10.1126/sciadv.adw1553 40644541 PMC12248311

[71] Pan J Li Y Gao W Jiang Q Geng L Ding J . Transcription factor Sp1 transcriptionally enhances GSDME expression for pyroptosis. Cell Death Dis. (2024) 15. doi: 10.1038/s41419-024-06455-6 38238307 PMC10796635

[72] Gustafsson ÅB Dorn GW . Evolving and expanding the roles of mitophagy as a homeostatic and pathogenic process. Physiol Rev. (2019) 99:853–92. doi: 10.1152/physrev.00005.2018 30540226 PMC6442924

[73] Murphy MP . How mitochondria produce reactive oxygen species. Biochem J. (2009) 417:1–13. 10.1042/BJ20081386 PMC2605959 19061483

[74] Shi W Zhou Q Lu L Zhang Y Zhang H Pu Y . Copper induced cytosolic escape of mitochondrial DNA and activation of cGAS-STING-NLRP3 pathway-dependent pyroptosis in C8-D1A cells. Ecotoxicol Environ Saf. (2024) 285:117085. doi: 10.1016/j.ecoenv.2024.117085 39321529

[75] Pétrilli V Papin S Dostert C Mayor A Martinon F Tschopp J . Activation of the NALP3 inflammasome is triggered by low intracellular potassium concentration. Cell Death Differ. (2007) 14:1583–9. doi: 10.1038/sj.cdd.4402195 17599094

[76] Cruz CM Rinna A Forman HJ Ventura AL Persechini PM Ojcius DM . ATP activates a reactive oxygen species-dependent oxidative stress response and secretion of proinflammatory cytokines in macrophages. J Biol Chem. (2007) 282:2871–9. doi: 10.1074/jbc.m608083200 17132626 PMC2693903

[77] Dostert C Pétrilli V Van Bruggen R Steele C Mossman BT Tschopp J . Innate immune activation through Nalp3 inflammasome sensing of asbestos and silica. Science. (2008) 320:674–7. doi: 10.1126/science.1156995 18403674 PMC2396588

[78] Zhou R Yazdi AS Menu P Tschopp J . A role for mitochondria in NLRP3 inflammasome activation. Nature. (2011) 469:221–5. doi: 10.1038/nature10156 21124315

[79] Su L Zhang J Gomez H Kellum JA Peng Z . Mitochondria ROS and mitophagy in acute kidney injury. Autophagy. (2023) 19:401–14. doi: 10.1080/15548627.2022.2084862 PMC9851232 35678504

[80] Zhang W Li G Luo R Lei J Song Y Wang B . Cytosolic escape of mitochondrial DNA triggers cGAS-STING-NLRP3 axis-dependent nucleus pulposus cell pyroptosis. Exp Mol Med. (2022) 54:129–42. doi: 10.1038/s12276-022-00729-9 35145201 PMC8894389

[81] Mun Y Kim J Choi YJ Lee BH . cGAS–STING–NF-κB axis mediates rotenone-induced NLRP3 inflammasome activation through mitochondrial DNA release. Antioxidants. (2025) 14:1276. doi: 10.3390/antiox14111276 41300433 PMC12649239

[82] Liu F Su Y Liu X Zhao L Wu Z Liu Y . Cell-free DNA: a metabolic byproduct with diagnostic and prognostic potential in rheumatic disorders. Front Pharmacol. (2025) 16. doi: 10.3389/fphar.2025.1537934 40008123 PMC11850341

[83] Liu F Yang Y Peng W Zhao N Chen J Xu Z . Mitophagy-promoting miR-138-5p promoter demethylation inhibits pyroptosis in sepsis-associated acute lung injury. Inflammation Res. (2023) 72:329–46. doi: 10.1007/s00011-022-01675-y 36538076

[84] Lee HM Yuk JM Kim KH Jang J Kang G Park JB . Mycobacterium abscessus activates the NLRP3 inflammasome via Dectin‐1–Syk and p62/SQSTM1. Immunol Cell Biol. (2012) 90:601–10. doi: 10.1038/icb.2011.72 PMC3389799 21876553

[85] Brough D Le Feuvre RA Wheeler RD Solovyova N Hilfiker S Rothwell NJ . Ca2+ stores and Ca2+ entry differentially contribute to the release of IL-1β and IL-1α from murine macrophages. J Immunol. (2003) 170:3029–36. doi: 10.4049/jimmunol.170.6.3029 12626557

[86] Chu J Thomas LM Watkins SC Franchi L Núñez G Salter RD . Cholesterol-dependent cytolysins induce rapid release of mature IL-1β from murine macrophages in a NLRP3 inflammasome and cathepsin B-dependent manner. J Leukocyte Biol. (2009) 86:1227–38. doi: 10.1189/jlb.0309164 19675207 PMC2774880

[87] Feldmeyer L Keller M Niklaus G Hohl D Werner S Beer HD . The inflammasome mediates UVB-induced activation and secretion of interleukin-1β by keratinocytes. Curr Biol. (2007) 17:1140–5. doi: 10.1016/j.cub.2007.05.074 17600714

[88] Lee GS Subramanian N Kim AI Aksentijevich I Goldbach-Mansky R Sacks DB . The calcium-sensing receptor regulates the NLRP3 inflammasome through Ca2+ and cAMP. Nature. (2012) 492:123–7. doi: 10.1038/nature11588 23143333 PMC4175565

[89] Rossol M Pierer M Raulien N Quandt D Meusch U Rothe K . Extracellular Ca2+ is a danger signal activating the NLRP3 inflammasome through G protein-coupled calcium sensing receptors. Nat Commun. (2012) 3:1329. doi: 10.1038/ncomms2339 23271661 PMC3535422

[90] Murakami T Ockinger J Yu J . Critical role for calcium mobilization in activation of the NLRP3 inflammasome. Proc Natl Acad Sci. (2012) 109:11282–7. doi: 10.1073/pnas.1117765109 22733741 PMC3396518

[91] Oleinik N Albayram O Kassir MF Atilgan FC Walton C Karakaya E . Alterations of lipid‐mediated mitophagy result in aging‐dependent sensorimotor defects. Aging Cell. (2023) 22. doi: 10.1111/acel.13954 37614052 PMC10577547

[92] Zhao S Zhang Y Li C Zheng Z Ma M . Succinate-mediated Ufsp2 transcription promotes high glucose-stimulated pyroptosis in rat retinal Müller cells by activating NLRP3 inflammasome. Biochem Biophys Res Commun. (2025) 783:152614. doi: 10.1016/j.bbrc.2025.152614 40946554

[93] Li M Wu W Hu B Chen H Wang J Zhang S . Atraric acid activates the FoxO3a/PINK1/Parkin signaling pathway to suppress chronic intermittent hypoxia-induced cardiac oxidative stress, inflammatory responses, and NLRP3 inflammasome activation. Chem Biol Interact. (2025) 421. doi: 10.1016/j.cbi.2025.111773 41093125

[94] Fan X Wu L Wang F Liu D Cen X Xia H . Mitophagy regulates kidney diseases. Kidney Dis. (2024), 1–15. doi: 10.1159/000541486 39664332 PMC11631111

[95] Wu BS Xiang HQ Yu YW Liu S Song DY Wu C . 3,4-benzo[a]pyrene aggravates myocardial infarction injury by activating NLRP3-related pyroptosis through PINK1/Parkin-mitophagy-mPTP opening axis. Int Immunopharmacol. (2023) 122:110481. doi: 10.1016/j.intimp.2023.110481 37390647

[96] She R Liu D Liao J Wang G Ge J Mei Z . Mitochondrial dysfunctions induce PANoptosis and ferroptosis in cerebral ischemia/reperfusion injury: from pathology to therapeutic potential. Front Cell Neurosci. (2023) 17. doi: 10.3389/fncel.2023.1191629 37293623 PMC10244524

[97] Xu Y Shen J Ran Z . Emerging views of mitophagy in immunity and autoimmune diseases. Autophagy. (2020) 16:3–17. doi: 10.1080/15548627.2019.1603547 PMC6984455 30951392

[98] Davidescu M Mezzasoma L Fettucciari K Pascucci L Pariano M Di Michele A . Cardiolipin-mediated temporal response to hydroquinone toxicity in human retinal pigmented epithelial cell line. Biochim Biophys Acta (BBA) - Mol Cell Res. (2023) 1870:119554. doi: 10.1016/j.bbamcr.2023.119554 37524263

[99] Chen W Zhao H Li Y . Mitochondrial dynamics in health and disease: mechanisms and potential targets. Signal Transduction Targeted Ther. (2023) 8. doi: 10.1038/s41392-023-01547-9 37669960 PMC10480456

[100] Vringer E Tait SWG . Mitochondria and cell death-associated inflammation. Cell Death Differ. (2023) 30:304–12. doi: 10.1038/s41418-022-01094-w PMC9950460 36447047

[101] Ouyang W Wang S Yan D Wu J Zhang Y Li W . The cGAS-STING pathway-dependent sensing of mitochondrial DNA mediates ocular surface inflammation. Signal Transduction Targeted Ther. (2023) 8. doi: 10.1038/s41392-023-01624-z 37735446 PMC10514335

[102] Xian H Watari K Sanchez-Lopez E Offenberger J Onyuru J Sampath H . Oxidized DNA fragments exit mitochondria via mPTP- and VDAC-dependent channels to activate NLRP3 inflammasome and interferon signaling. Immunity. (2022) 55:1370–1385.e8. doi: 10.1016/j.immuni.2022.06.007 35835107 PMC9378606

[103] Miao R Jiang C Chang WY Zhang H An J Ho F . Gasdermin D permeabilization of mitochondrial inner and outer membranes accelerates and enhances pyroptosis. Immunity. (2023) 56:2523–2541 e8. doi: 10.1016/j.immuni.2023.10.004 37924812 PMC10872579

[104] Huang Y Yang L . Regulation of pyroptosis and ferroptosis by mitophagy in chronic kidney disease. (2024). 10.11817/j.issn.1672-7347.2024.240458 PMC11964809 40177760

[105] He X Qian Y Li Z Fan EK Li Y Wu L . TLR4-upregulated IL-1β and IL-1RI promote alveolar macrophage pyroptosis and lung inflammation through an autocrine mechanism. Sci Rep. (2016) 6:31663. doi: 10.1038/srep31663 27526865 PMC4985817

[106] Zhou Q Zhang Y Lu L Zhang H Zhao C Pu Y . Copper induces microglia-mediated neuroinflammation through ROS/NF-κB pathway and mitophagy disorder. Food Chem Toxicol. (2022) 168:113369. doi: 10.1016/j.fct.2022.113369 35985369

[107] Wu M Yu C Wen F Li Y Zhang X Wang Y . NLRP3 inflammasome inhibits mitophagy during the progression of temporal lobe epilepsy. Sci Rep. (2025) 15. doi: 10.1038/s41598-025-01087-y 40348802 PMC12065917

[108] Liu R Liu Y Liu C Gao A Wang L Tang H . NEK7-mediated activation of NLRP3 inflammasome is coordinated by potassium efflux/Syk/JNK signaling during Staphylococcus aureus infection. Front Immunol. (2021) 12. doi: 10.3389/fimmu.2021.747370 34603335 PMC8481599

[109] Zhang T Ding S Wang R . Research progress of mitochondrial mechanism in NLRP3 inflammasome activation and exercise regulation of NLRP3 inflammasome. Int J Mol Sci. (2021) 22:10866. doi: 10.3390/ijms221910866 34639204 PMC8509472

[110] Weber ANR McManus RM Hornung V Geyer M Kuemmerle-Deschner JB Latz E . The expanding role of the NLRP3 inflammasome from periodic fevers to therapeutic targets. Nat Immunol. (2025) 26:1453–66. doi: 10.1038/s41590-025-02230-7 40826276

[111] Lv S Liu H Wang H . The interplay between autophagy and NLRP3 inflammasome in ischemia/reperfusion injury. Int J Mol Sci. (2021) 22:8773. doi: 10.3390/ijms22168773 34445481 PMC8395601

[112] Wei Y You Y Zhang J Ban J Min H Li C . Crystalline silica-induced macrophage pyroptosis interacting with mitophagy contributes to pulmonary fibrosis via modulating mitochondria homeostasis. J Hazard Mater. (2023) 454:131562. doi: 10.1016/j.jhazmat.2023.131562 37148789

[113] Zhou F Lian W Yuan X Wang Z Xia C Yan Y . Cornuside alleviates cognitive impairments induced by Aβ1-42 through attenuating NLRP3-mediated neurotoxicity by promoting mitophagy. Alzheimer's Res Ther. (2025) 17. doi: 10.1186/s13195-025-01695-w 39972387 PMC11837312

[114] Wu D Zhang H Wu Q Li F Wang Y Liu S . Sestrin 2 protects against LPS-induced acute lung injury by inducing mitophagy in alveolar macrophages. Life Sci. (2021) 267. doi: 10.1016/j.lfs.2020.118941 33359748

[115] Chen K Feng L Hu W Chen J Wang X Wang L . Optineurin inhibits NLRP3 inflammasome activation by enhancing mitophagy of renal tubular cells in diabetic nephropathy. FASEB J. (2018) 33:4571–85. doi: 10.1096/fj.201801749rrr 30571313

[116] Xu Y Wang J Xu W Ding F Ding W . Prohibitin 2-mediated mitophagy attenuates renal tubular epithelial cells injury by regulating mitochondrial dysfunction and NLRP3 inflammasome activation. Am J Physiol Renal Physiol. (2019) 316:F396–407. doi: 10.1152/ajprenal.00420.2018 30539655

[117] Wang S Long H Hou L Feng B Ma Z Wu Y . The mitophagy pathway and its implications in human diseases. Signal Transduction Targeted Ther. (2023) 8. doi: 10.1038/s41392-023-01503-7 37582956 PMC10427715

[118] Kazlauskaite A Kondapalli C Gourlay R Campbell DG Ritorto MS Hofmann K . Parkin is activated by PINK1-dependent phosphorylation of ubiquitin at Ser65. Biochem J. (2014) 460:127–41. doi: 10.1042/bj20140334 24660806 PMC4000136

[119] Peng X Zhang C Zhou ZM Wang K Gao JW Qian ZY . A20 attenuates pyroptosis and apoptosis in nucleus pulposus cells via promoting mitophagy and stabilizing mitochondrial dynamics. Inflammation Res. (2022) 71:695–710. doi: 10.1007/s00011-022-01570-6 35426501

[120] Peng X Zhang C Gao JW Wang F Bao JP Zhou ZM . A20 ameliorates disc degeneration by suppressing mTOR/BNIP3 axis-mediated mitophagy. Genes Genomics. (2022) 45:657–71. doi: 10.1007/s13258-022-01343-9 36583816

[121] Yu J Nagasu H Murakami T Hoang H Broderick L Hoffman HM . Inflammasome activation leads to Caspase-1–dependent mitochondrial damage and block of mitophagy. Proc Natl Acad Sci. (2014) 111:15514–9. doi: 10.1073/pnas.1414859111 25313054 PMC4217429

[122] Meng Z Gao M Wang C Guan S Zhang D Lu J . Apigenin alleviated high-fat-diet-induced hepatic pyroptosis by mitophagy-ROS-CTSB-NLRP3 pathway in mice and AML12 cells. J Agric Food Chem. (2023) 71:7032–45. doi: 10.1021/acs.jafc.2c07581 37141464

[123] Radulovic M Yang C Stenmark H . Lysosomal membrane homeostasis and its importance in physiology and disease. Nat Rev Mol Cell Biol. (2025) 27:71–87. doi: 10.1038/s41580-025-00873-w 40759742

[124] Lin Q Li S Jiang N Shao X Zhang M Jin H . PINK1-parkin pathway of mitophagy protects against contrast-induced acute kidney injury via decreasing mitochondrial ROS and NLRP3 inflammasome activation. Redox Biol. (2019) 26. doi: 10.1016/j.redox.2019.101254 31229841 PMC6597739

[125] Mouton-Liger F Rosazza T Sepulveda-Diaz J Ieang A Hassoun SM Claire E . Parkin deficiency modulates NLRP3 inflammasome activation by attenuating an A20‐dependent negative feedback loop. Glia. (2018) 66:1736–51. 10.1002/glia.23337 PMC6190839 29665074

[126] Wang Z Chan SW Zhao H Miu KK Chan WY . Outlook of PINK1/Parkin signaling in molecular etiology of Parkinson’s disease, with insights into Pink1 knockout models. Zool Res. (2023) 44:559–76. doi: 10.24272/j.issn.2095-8137.2022.406 PMC10236303 37161651

[127] Tian L Li N Li K Tan Y Han J Lin B . Ambient ozone exposure induces ROS related-mitophagy and pyroptosis via NLRP3 inflammasome activation in rat lung cells. Ecotoxicol Environ Saf. (2022) 240:113663. doi: 10.1016/j.ecoenv.2022.113663 35642860

[128] Li J Li M Wang R Lan J Yu L Gao J . Mitophagy protects against silver nanoparticle–induced hepatotoxicity by inhibiting mitochondrial ROS and the NLRP3 inflammasome. Ecotoxicol Environ Saf. (2024) 273:116137. doi: 10.1016/j.ecoenv.2024.116137 38417314

[129] Martin NR Fairn GD . Straight A’s: protein S-acylation in the activation and autophagic degradation of NOD-like receptors. Biochem Soc Trans. (2025) 53:751–61. doi: 10.1042/bst20253026 40613780 PMC12326153

[130] Lupfer C Thomas PG Anand PK Vogel P Milasta S Martinez J . Receptor interacting protein kinase 2–mediated mitophagy regulates inflammasome activation during virus infection. Nat Immunol. (2013) 14:480–8. doi: 10.1038/ni.2563 23525089 PMC3631456

[131] Sun G Su W Bao J Sun G Su W Bao J . Dietary full-fat rice bran prevents the risk of heart ferroptosis and imbalance of energy metabolism induced by prolonged cold stimulation. Food Funct. (2023) 14:1530–44. doi: 10.1039/d2fo03673h 36655680

[132] Wang X Shen Z Zhang H Zhang HJ Li F Yu L . Bexarotene improves motor function after spinal cord injury in mice. Neural Regener Res. (2023) 18:2733–42. doi: 10.4103/1673-5374.373676 37449638 PMC10358692

[133] Goswami AB Karadarević D Castaño-Rodríguez N . Immunity-related GTPase IRGM at the intersection of autophagy, inflammation, and tumorigenesis. Inflammation Res. (2022) 71:785–95. doi: 10.1007/s00011-022-01595-x 35699756 PMC9192921

[134] Wang DX Dong ZJ Deng SX Tian YM Xiao YJ Li X . GDF11 slows excitatory neuronal senescence and brain ageing by repressing p21. Nat Commun. (2023) 14. doi: 10.1038/s41467-023-43292-1 37978295 PMC10656444

[135] Lin Q Li S Jiang N Jin H Shao X Zhu X . Inhibiting NLRP3 inflammasome attenuates apoptosis in contrast-induced acute kidney injury through the upregulation of HIF1A and BNIP3-mediated mitophagy. Autophagy. (2020) 17:2975–90. doi: 10.1080/15548627.2020.1848971 33345685 PMC8525960

[136] Yan B Ju W Xue H Xu Z Qiu D Tan G . The hidden driver of osteoporosis: mitochondria-dominated inflammation. Cell Commun Signaling. (2025) 23. doi: 10.1186/s12964-025-02453-z 41126358 PMC12541965

[137] Ma GD Liu YH Zhang QL Zhang BG Zhao N Wang QL . Pre-endurance training prevents acute alcoholic liver injury in rats through the regulation of damaged mitochondria accumulation and mitophagy balance. Hepatol Int. (2014) 8:425–35. doi: 10.1007/s12072-014-9529-5 26202644

[138] Hong Y Zhou X Li Q Chen J Wei Y Long C . X-box binding protein 1 caused an imbalance in pyroptosis and mitophagy in immature rats with di-(2-ethylhexyl) phthalate-induced testis toxicity. Genes Dis. (2024) 11:935–51. doi: 10.1016/j.gendis.2023.02.030 37692514 PMC10491871

[139] Luo G Chen L Chen M Mao L Zeng Q Zou Y . Hirudin inhibit the formation of NLRP3 inflammasome in cardiomyocytes via suppressing oxidative stress and activating mitophagy. Heliyon. (2024) 10. doi: 10.1016/j.heliyon.2023.e23077 38163129 PMC10754874

[140] Wang W Gao W Zhang L Xia Z Zhao B . SNAP25 ameliorates postoperative cognitive dysfunction by facilitating PINK1-dependent mitophagy and impeding caspase-3/GSDME-dependent pyroptosis. Exp Neurol. (2023) 367. doi: 10.1016/j.expneurol.2023.114463 37295545

[141] Ding HG Li Y Li XS Liu XQ Wang KR Wen MY . Hypercapnia promotes microglial pyroptosis via inhibiting mitophagy in hypoxemic adult rats. CNS Neurosci Ther. (2020) 26:1134–46. doi: 10.1111/cns.13435 32666671 PMC7564198

[142] Wang W Zhao B Gao W Song W Hou J Zhang L . Inhibition of PINK1-mediated mitophagy contributes to postoperative cognitive dysfunction through activation of caspase-3/GSDME-dependent pyroptosis. ACS Chem Neurosci. (2023) 14:1249–60. doi: 10.1021/acschemneuro.2c00691 36946264

[143] Yu W Lyu J Jia L Sheng M Yu H Du H . Dexmedetomidine ameliorates hippocampus injury and cognitive dysfunction induced by hepatic ischemia/reperfusion by activating SIRT3-mediated mitophagy and inhibiting activation of the NLRP3 inflammasome in young rats. Oxid Med Cell Longevity. (2020) 2020:1–17. doi: 10.1155/2020/7385458 34493950 PMC8418694

[144] Hu Z Yuan Y Zhang X Lu Y Dong N Jiang X . Human umbilical cord mesenchymal stem cell‐derived exosomes attenuate oxygen‐glucose deprivation/reperfusion‐induced microglial pyroptosis by promoting FOXO3a‐dependent mitophagy. Oxid Med Cell Longevity. (2021) 2021. doi: 10.1155/2021/6219715 34765084 PMC8577931

[145] Jiao K Lai Z Cheng Q Yang Z Liao W Liao Y . Glycosides of Buyang Huanwu decoction inhibits inflammation associated with cerebral ischemia-reperfusion via the PINK1/Parkin mitophagy pathway. J Ethnopharmacol. (2024) 325:117766. doi: 10.1016/j.jep.2024.117766 38266949

[146] Wang W Gao W Gong P Song W Bu X Hou J . Neuronal-specific TNFAIP1 ablation attenuates postoperative cognitive dysfunction via targeting SNAP25 for K48-linked ubiquitination. Cell Commun Signaling. (2023) 21. doi: 10.1186/s12964-023-01390-z 38102610 PMC10722859

[147] Ye Y Ren K Dong Y Yang L Zhang D Yuan Z . Mitochondria-targeting pyroptosis amplifier of lonidamine-modified black phosphorus nanosheets for glioblastoma treatments. ACS Appl Materials Interfaces. (2023) 15:26285–97. doi: 10.1021/acsami.3c01559 37220137

[148] Tan Z Dong F Wu L Feng Y Zhang M Zhang F . Transcutaneous electrical nerve stimulation (TENS) alleviates brain ischemic injury by regulating neuronal oxidative stress, pyroptosis, and mitophagy. Mediators Inflammation. (2023) 2023:1–13. doi: 10.1155/2023/5677865 37101593 PMC10125764

[149] He Q Li Z Meng C Wu J Zhao Y Zhao J . Parkin-dependent mitophagy is required for the inhibition of ATF4 on NLRP3 inflammasome activation in cerebral ischemia-reperfusion injury in rats. Cells. (2019) 8:897. doi: 10.3390/cells8080897 31416289 PMC6721752

[150] Younossi Z Tacke F Arrese M Chander Sharma B Mostafa I Bugianesi E . Global perspectives on nonalcoholic fatty liver disease and nonalcoholic steatohepatitis. Hepatology. (2019) 69:2672–82. doi: 10.1002/hep.30251 30179269

[151] Szabo G Petrasek J . Inflammasome activation and function in liver disease. Nat Rev Gastroenterol Hepatol. (2015) 12:387–400. doi: 10.1038/nrgastro.2015.94 26055245

[152] Gao X Ruan Y Zhu X Lin X Xin Y Li X . Deoxycholic acid promotes pyroptosis in free fatty acid-induced steatotic hepatocytes by inhibiting PINK1-mediated mitophagy. Inflammation. (2022) 45:639–50. doi: 10.1007/s10753-021-01573-1 34674097

[153] Yu X Hao M Liu Y Ma X Lin W Xu Q . Liraglutide ameliorates non-alcoholic steatohepatitis by inhibiting NLRP3 inflammasome and pyroptosis activation via mitophagy. Eur J Pharmacol. (2019) 864:172715. doi: 10.1016/j.ejphar.2019.172715 31593687

[154] Zhang Q Hu J Mao A Liu M Guan S Lu J . Ginsenoside Rb1 alleviated concanavalin A-induced hepatocyte pyroptosis by activating mitophagy. Food Funct. (2023) 14:3793–803. doi: 10.1039/d2fo03130b 37000511

[155] Zhang H Shi H Xie W Meng M Wang Y Ma N . Subacute ruminal acidosis induces pyroptosis via the mitophagy-mediated NLRP3 inflammasome activation in the livers of dairy cows fed a high-grain diet. J Dairy Sci. (2024) 107:4092–107. doi: 10.3168/jds.2023-23718 38278294

[156] Eid N Ito Y Horibe A Otsuki Y . Ethanol-induced mitophagy in liver is associated with activation of the PINK1-Parkin pathway triggered by oxidative DNA damage. Histol Histopathol. (2016). 10.14670/HH-11-747 26935412

[157] Gaul S Leszczynska A Alegre F Kaufmann B Johnson CD Adams LA . Hepatocyte pyroptosis and release of inflammasome particles induce stellate cell activation and liver fibrosis. J Hepatol. (2021) 74:156–67. doi: 10.1016/j.jhep.2020.07.041 32763266 PMC7749849

[158] Liu Z Wang M Wang X Bu Q Wang Q Su W . XBP1 deficiency promotes hepatocyte pyroptosis by impairing mitophagy to activate mtDNA-cGAS-STING signaling in macrophages during acute liver injury. Redox Biol. (2022) 52:102305. doi: 10.1016/j.redox.2022.102305 35367811 PMC8971356

[159] Zhang R Guan S Meng Z Zhang D Lu J . Ginsenoside Rb1 alleviates 3-MCPD-induced renal cell pyroptosis by activating mitophagy. Food Chem Toxicol. (2024) 186. doi: 10.1016/j.fct.2024.114522 38373586

[160] Zhang C Song Y Chen L Chen P Yuan M Meng Y . Urolithin A attenuates hyperuricemic nephropathy in fructose-fed mice by impairing STING-NLRP3 axis-mediated inflammatory response via restoration of Parkin-dependent mitophagy. Front Pharmacol. (2022) 13. doi: 10.3389/fphar.2022.907209 35784701 PMC9240289

[161] Li Y Hu C Zhai P Zhang J Jiang J Suo J . Fibroblastic reticular cell-derived exosomes are a promising therapeutic approach for septic acute kidney injury. Kidney Int. (2024) 105:508–23. doi: 10.1016/j.kint.2023.12.007 38163633

[162] Guo J Wang R Liu D . Bone marrow-derived mesenchymal stem cells ameliorate sepsis-induced acute kidney injury by promoting mitophagy of renal tubular epithelial cells via the SIRT1/Parkin axis. Front Endocrinol. (2021) 12. doi: 10.3389/fendo.2021.639165 34248837 PMC8267935

[163] Tan S Chen S . Macrophage autophagy and silicosis: current perspective and latest insights. Int J Mol Sci. (2021) 22:453. doi: 10.3390/ijms22010453 33466366 PMC7795780

[164] Benmerzoug S Rose S Bounab B Gosset D Duneau L Chenuet P . STING-dependent sensing of self-DNA drives silica-induced lung inflammation. Nat Commun. (2018) 9. doi: 10.1038/s41467-018-07425-1 30523277 PMC6283886

[165] Gong T Fu Y Wang Q Loughran PA Li Y Billiar TR . Decoding the multiple functions of ZBP1 in the mechanism of sepsis-induced acute lung injury. Commun Biol. (2024) 7. doi: 10.1038/s42003-024-07072-x 39433574 PMC11493966

[166] Lyu JJ Mehta JL Li Y Ye L Sun SN Sun HS . Mitochondrial autophagy and NLRP3 inflammasome in pulmonary tissues from severe combined immunodeficient mice after cardiac arrest and cardiopulmonary resuscitation. Chin Med J. (2018) 131:1174–84. doi: 10.4103/0366-6999.231519 29722336 PMC5956768

[167] Mohsin M Zaki A Tabassum G Khan S Ali S Ahmad T . Urolithin-A supplementation alleviates sepsis-induced acute lung injury by reducing mitochondrial dysfunction and modulating macrophage polarization. Mitochondrion. (2025) 84:102047. doi: 10.1016/j.mito.2025.102047 40328344

[168] Lao Z Fang X Shen S Zhang Y Chen X Zhang H . The onset of systemic lupus erythematosus triggers nucleus pulposus cell pyroptosis to exacerbate intervertebral disc degeneration. J Inflammation Res. (2024) 17:7705–19. doi: 10.2147/jir.s486297 39473981 PMC11520921

[169] Dong Z Yang B Jia M Yang C Wang S Mu H . DDIT3/CHOP promotes LPS/ATP-induced pyroptosis in osteoblasts via mitophagy inhibition. Biochim Biophys Acta (BBA) - Mol Cell Res. (2024) 1871:119712. doi: 10.1016/j.bbamcr.2024.119712 38521466

[170] Hong Z Wang H Zhang T Xu L Zhai Y Zhang X . The HIF-1/ BNIP3 pathway mediates mitophagy to inhibit the pyroptosis of fibroblast-like synoviocytes in rheumatoid arthritis. Int Immunopharmacol. (2024) 127:111378. doi: 10.1016/j.intimp.2023.111378 38141408

[171] Ouyang X Chen X Xin F Sha P Tong S Liu M . p21 improving osteoarthritis by regulating the pyroptosis of cartilage cells. Iranian J Public Health. (2024). doi: 10.18502/ijph.v53i3.15146 38919298 PMC11194650

[172] Liu K Wang H Wang Y Zhang X Wang R Zhang Z . Exploring the therapeutic potential of Sirt6-enriched adipose stem cell-derived exosomes in myocardial ischemia–reperfusion injury: unfolding new epigenetic frontiers. Clin Epigenet. (2024) 16. doi: 10.1186/s13148-023-01618-2 38172884 PMC10765803

[173] Lagunas-Rangel FA . Sirtuins in mitophagy: key gatekeepers of mitochondrial quality. Mol Cell Biochem. (2025) 480:5877–96. doi: 10.1007/s11010-025-05358-0 40705152 PMC12594717

[174] Yu X Gao J Zhang C . Sepsis-induced cardiac dysfunction: mitochondria and energy metabolism. Intensive Care Med Exp. (2025) 13. doi: 10.1186/s40635-025-00728-w 39966268 PMC11836259

[175] Song C Zhang Y Pei Q Zheng L Wang M Shi Y . HSP70 alleviates sepsis-induced cardiomyopathy by attenuating mitochondrial dysfunction-initiated NLRP3 inflammasome-mediated pyroptosis in cardiomyocytes. Burns Trauma. (2022) 10. doi: 10.1093/burnst/tkac043 36439706 PMC9684341

[176] Xu H Guo H Tang Z Hao R Wang S Jin P . Follistatin‐like 1 protects against doxorubicin‐induced cardiotoxicity by preventing mitochondrial dysfunction through the SIRT6/Nrf2 signaling pathway. Cell Biol Int. (2024) 48:795–807. doi: 10.1002/cbin.12147 38436106

[177] Wang Q Shi H Zhuang H Dong G Gao K Liu L . SIRT6 mitigates doxorubicin-induced cardiomyopathy via amelioration of mitochondrial dysfunction: a mechanistic study implicating the activation of the Nrf-2/FUNDC1 signaling axis. Int J Med Sci. (2025) 22:1640–57. doi: 10.7150/ijms.101520 40093797 PMC11905259

[178] Yumnamcha T Devi TS Singh LP . Auranofin mediates mitochondrial dysregulation and inflammatory cell death in human retinal pigment epithelial cells: implications of retinal neurodegenerative diseases. Front Neurosci. (2019) 13. doi: 10.3389/fnins.2019.01065 31649499 PMC6795687

[179] Singh LP . The role of Txnip in mitophagy dysregulation and inflammasome activation in diabetic retinopathy: a new perspective. JOJ Ophthalmol. (2017) 4. doi: 10.19080/jojo.2017.04.555643 29376145 PMC5786434

[180] Ren K Li X . MCC950 targets the ROS-NEK7-NLRP3 axis to improve type 2 diabetic retinopathy. Sci Rep. (2025) 15. doi: 10.1038/s41598-025-18438-4 40987781 PMC12457656

[181] Sun Y Lv D Xie Y Xu H Li X Li F . PINK1-mediated mitophagy induction protects against preeclampsia by decreasing ROS and trophoblast pyroptosis. Placenta. (2023) 143:1–11. doi: 10.1016/j.placenta.2023.09.010 37788592

[182] Diao L Liu W Xu Y Zhang B Meng L Yin R . New insights into micro-algal astaxanthin's effect on deoxynivalenol-induced spleen lymphocytes pyroptosis in Cyprinus carpio: involving mitophagy and mtROS-NF-κB-dependent NLRP3 inflammasome. Fish Shellfish Immunol. (2024) 144:109259. doi: 10.1016/j.fsi.2023.109259 38040132

[183] Chen Y Yuan C Qin W Yu B Wei D Wu P . TMAO promotes vascular endothelial cell pyroptosis via the LPEAT-mitophagy pathway. Biochem Biophys Res Commun. (2024) 703:149667. doi: 10.1016/j.bbrc.2024.149667 38382362

[184] Hou M Chen F He Y Tan Z Han X Shi Y . Dexmedetomidine against intestinal ischemia/reperfusion injury: a systematic review and meta-analysis of preclinical studies. Eur J Pharmacol. (2023) 959:176090. doi: 10.1016/j.ejphar.2023.176090 37778612

[185] Dai X Sun F Deng K Lin G Yin W Chen H . Mallotucin D, a clerodane diterpenoid from Croton crassifolius, suppresses HepG2 cell growth via inducing autophagic cell death and pyroptosis. Int J Mol Sci. (2022) 23. doi: 10.3390/ijms232214217 36430694 PMC9698996

[186] Kim SY Song X Zhang L Bartlett DL Lee YJ . Role of Bcl-xL/Beclin-1 in interplay between apoptosis and autophagy in oxaliplatin and bortezomib-induced cell death. Biochem Pharmacol. (2014) 88:178–88. doi: 10.1016/j.bcp.2014.01.027 24486574 PMC3969586

[187] Li W Cai Z Schindler F Afjehi-Sadat L Montsch B Heffeter P . Elevated PINK1/Parkin-dependent mitophagy and boosted mitochondrial function mediate protection of HepG2 cells from excess palmitic acid by hesperetin. J Agric Food Chem. (2024) 72:13039–53. doi: 10.1021/acs.jafc.3c09132 38809522 PMC11181321

[188] Liu S Wang L Zhu L Zhao T Han P Yan F . Mechanism and regulation of mitophagy in liver diseases: a review. Front Cell Dev Biol. (2025) 13. doi: 10.3389/fcell.2025.1614940 40655944 PMC12246547

[189] Davidson SM Adameová A Barile L Cabrera-Fuentes HA Lazou A Pagliaro P . Mitochondrial and mitochondrial‐independent pathways of myocardial cell death during ischaemia and reperfusion injury. J Cell Mol Med. (2020) 24:3795–806. doi: 10.1111/jcmm.15127 32155321 PMC7171390

[190] Zhu Y . Inhibition of PINK1 senses ROS signaling to facilitate neuroblastoma cell pyroptosis. Autophagy. (2025) 21:2091–110. doi: 10.1080/15548627.2025.2487037 40160153 PMC12459364

[191] Liu M Lu J Yang S Chen Y Yu J Guan S . Alliin alleviates LPS-induced pyroptosis via promoting mitophagy in THP-1 macrophages and mice. Food Chem Toxicol. (2022) 160:112811. doi: 10.1016/j.fct.2022.112811 34999177

[192] Feng WD Wang Y Luo T Jia X Cheng CQ Wang HJ . Scoparone suppresses mitophagy-mediated NLRP3 inflammasome activation in inflammatory diseases. Acta Pharmacol Sin. (2023) 44:1238–51. doi: 10.1038/s41401-022-01028-9 PMC10203299 36522512

[193] Han X Xu T Fang Q Zhang H Yue L Hu G . Quercetin hinders microglial activation to alleviate neurotoxicity via the interplay between NLRP3 inflammasome and mitophagy. Redox Biol. (2021) 44:102010. doi: 10.1016/j.redox.2021.102010 34082381 PMC8182123

[194] Cheng Y Lu Z Mao T Song Y Qu Y Chen X . Magnoflorine ameliorates chronic kidney disease in high-fat and high-fructose-fed mice by promoting Parkin/PINK1-dependent mitophagy to inhibit NLRP3/Caspase-1-mediated pyroptosis. J Agric Food Chem. (2024) 72:12775–87. doi: 10.1021/acs.jafc.3c09634 38776285

[195] Luo T J.X. Feng WD Wang JY Xie F Kong LD . Bergapten inhibits NLRP3 inflammasome activation and pyroptosis via promoting mitophagy. (2023). doi: 10.1038/s41401-023-01094-7 PMC10462717 37142684

[196] Yang SR Hsu WH Wu CY Shang HS Liu FC Chen A . Accelerated, severe lupus nephritis benefits from treatment with honokiol by immunoregulation and differentially regulating NF‐κB/NLRP3 inflammasome and sirtuin 1/autophagy axis. FASEB J. (2020) 34:13284–99. doi: 10.1096/fj.202001326r 32813287

[197] Li Y Li J Yang L Ren F Dong K Zhao Z . Ginsenoside Rb1 protects hippocampal neurons in depressed rats based on mitophagy-regulated astrocytic pyroptosis. Phytomedicine. (2023) 121:155083. doi: 10.1016/j.phymed.2023.155083 37722244

[198] Liu H Zhan X Xu G Wang Z Li R Wang Y . Cryptotanshinone specifically suppresses NLRP3 inflammasome activation and protects against inflammasome-mediated diseases. Pharmacol Res. (2021) 164:105384. doi: 10.1016/j.phrs.2020.105384 33352229

[199] Dong G Xu N Wang M Zhao Y Jiang F Bu H . Anthocyanin extract from purple sweet potato exacerbate mitophagy to ameliorate pyroptosis in Klebsiella pneumoniae infection. Int J Mol Sci. (2021) 22:11422. doi: 10.3390/ijms222111422 34768852 PMC8583717

[200] Wang X Yuan P Zeng M Sun M Wang X Zheng X . Allantoin derived from Dioscorea opposita Thunb ameliorates cyclophosphamide-induced premature ovarian failure in female rats by attenuating apoptosis, autophagy and pyroptosis. Cureus. (2023). doi: 10.7759/cureus.50351 38089953 PMC10713354

[201] Du S Li C Lu Y Lei X Zhang Y Li S . Dioscin alleviates crystalline silica-induced pulmonary inflammation and fibrosis through promoting alveolar macrophage autophagy. Theranostics. (2019) 9:1878–92. doi: 10.7150/thno.29682 31037145 PMC6485284

[202] Zhang Z Huang T Chen X Chen J Yuan H Yi N . Acetyl zingerone inhibits chondrocyte pyroptosis and alleviates osteoarthritis progression by promoting mitophagy through the PINK1/parkin signaling pathway. Int Immunopharmacol. (2025) 161:115055. doi: 10.1016/j.intimp.2025.115055 40494201

[203] Ding C Zhang C . Reducing myocardial infarction by combination of irisin and Dendrobium nobile Lindl through inhibiting Nod-like receptor protein-3-related pyroptosis and activating PINK1/Parkin-mitophagy during aging. Chin J Physiol. (2023) 66:351–8. doi: 10.4103/cjop.cjop-d-23-00032 37929346

[204] Chen L Mao M Liu D Liu W Wang Y Xie L . HC067047 as a potent TRPV4 inhibitor repairs endotoxemia colonic injury. Int Immunopharmacol. (2023) 116:109648. doi: 10.1016/j.intimp.2022.109648 36706595

[205] Zou J Li W Misra A Yue F Song K Chen Q . The viral restriction factor Tetherin prevents leucine-rich pentatricopeptide repeat-containing protein (LRPPRC) from association with Beclin 1 and B-cell CLL/lymphoma 2 (Bcl-2) and enhances autophagy and mitophagy. J Biol Chem. (2015) 290:7269–79. doi: 10.1074/jbc.m114.627679 25631043 PMC4358145

[206] She Y Shao L Jiao K Sun R Lang T Long H . Glycosides of Buyang Huanwu decoction inhibits pyroptosis associated with cerebral ischemia-reperfusion through Nrf2-mediated antioxidant signaling pathway both *in vivo* and *in vitro*. Phytomedicine. (2023) 120:155001. doi: 10.1016/j.phymed.2023.155001 37619321

[207] Miao C Zhao Y Chen Y Wang R Ren N Chen B . Investigation of He’s Yang Chao recipe against oxidative stress-related mitophagy and pyroptosis to improve ovarian function. Front Endocrinol. (2023) 14. doi: 10.3389/fendo.2023.1077315 36777359 PMC9911881

[208] Chen J Luo W Hu C Ren M Xu H Xu X . Tanshinone IIA analogue 15a inhibits NLRP3-mediated inflammation by activating mitophagy in macrophages to alleviate acute tubular necrosis. Int Immunopharmacol. (2023) 118:110065. doi: 10.1016/j.intimp.2023.110065 37004347

[209] Deng D Zhao M Liu H Zhou S Liu H You L . Xijiao Dihuang decoction combined with Yinqiao powder promotes autophagy-dependent ROS decrease to inhibit ROS/NLRP3/pyroptosis regulation axis in influenza virus infection. Phytomedicine. (2024) 128:155446. doi: 10.1016/j.phymed.2024.155446 38518643

[210] Ma Z Xie K Xue X Li J Yang Y Wu J . Si-Wu-Tang attenuates hepatocyte PANoptosis and M1 polarization of macrophages in non-alcoholic fatty liver disease by influencing the intercellular transfer of mtDNA. J Ethnopharmacol. (2024) 328:118057. doi: 10.1016/j.jep.2024.118057 38518965

[211] Xue D Zhang Y Shen A Tang T Liu B Lv L . mRNA therapy: A new frontier in regenerative medicine. Interdiscip Med. (2025) 3. doi: 10.1002/inmd.2024005442587375

[212] Chan A Tsourkas A . Intracellular protein delivery: Approaches, challenges, and clinical applications. BME Front. (2024) 5. doi: 10.34133/bmef.0035 38282957 PMC10809898

